# Phenotypic effects of genetic variants associated with autism

**DOI:** 10.1038/s41591-023-02408-2

**Published:** 2023-06-26

**Authors:** Thomas Rolland, Freddy Cliquet, Richard J. L. Anney, Clara Moreau, Nicolas Traut, Alexandre Mathieu, Guillaume Huguet, Jinjie Duan, Varun Warrier, Swan Portalier, Louise Dry, Claire S. Leblond, Elise Douard, Frédérique Amsellem, Simon Malesys, Anna Maruani, Roberto Toro, Anders D. Børglum, Jakob Grove, Simon Baron-Cohen, Alan Packer, Wendy K. Chung, Sébastien Jacquemont, Richard Delorme, Thomas Bourgeron

**Affiliations:** 1 Human Genetics and Cognitive Functions, Institut Pasteur, UMR3571 CNRS, IUF, Université Paris Cité, Paris, France; 2 Centre for Neuropsychiatric Genetics and Genomics, Division of Psychological Medicine and Clinical Neurosciences, Cardiff University, Cardiff, UK; 3 Center for Research and Interdisciplinarity (CRI), Université Paris Descartes, Paris, France; 4 Centre de Recherche du Centre Hospitalier Universitaire Sainte-Justine, Montréal, Québec Canada; 5 The Lundbeck Foundation Initiative for Integrative Psychiatric Research, iPSYCH, Aarhus, Denmark; 6 Department of Biomedicine and the iSEQ Centre, Aarhus University, Aarhus, Denmark; 7 Center for Genomics and Personalized Medicine, Aarhus, Denmark; 8 Autism Research Centre, Department of Psychiatry, University of Cambridge, Cambridge, UK; 9 Département de Pédiatrie, Université de Montréal, Montréal, Québec Canada; 10 Department of Child and Adolescent Psychiatry, Robert Debré Hospital, APHP, Paris, France; 11 Bioinformatics Research Centre, Aarhus University, Aarhus, Denmark; 12 Simons Foundation, New York, NY USA; 13 Department of Pediatrics, Columbia University Medical Center, New York, NY USA

**Keywords:** Genetics research, Autism spectrum disorders

## Abstract

While over 100 genes have been associated with autism, little is known about the prevalence of variants affecting them in individuals without a diagnosis of autism. Nor do we fully appreciate the phenotypic diversity beyond the formal autism diagnosis. Based on data from more than 13,000 individuals with autism and 210,000 undiagnosed individuals, we estimated the odds ratios for autism associated to rare loss-of-function (LoF) variants in 185 genes associated with autism, alongside 2,492 genes displaying intolerance to LoF variants. In contrast to autism-centric approaches, we investigated the correlates of these variants in individuals without a diagnosis of autism. We show that these variants are associated with a small but significant decrease in fluid intelligence, qualification level and income and an increase in metrics related to material deprivation. These effects were larger for autism-associated genes than in other LoF-intolerant genes. Using brain imaging data from 21,040 individuals from the UK Biobank, we could not detect significant differences in the overall brain anatomy between LoF carriers and non-carriers. Our results highlight the importance of studying the effect of the genetic variants beyond categorical diagnosis and the need for more research to understand the association between these variants and sociodemographic factors, to best support individuals carrying these variants.

## Abstract (web summary)

Analyses of data from more than 200,000 individuals identify phenotypic features associated to carrying variants in autism-associated genes, in individuals with and without a diagnosis of autism.

## Main

Autism is a heterogeneous condition characterized by atypical social communication, as well as unusually restricted or stereotyped interests^1^. Its genetic architecture is highly complex, with contributions from monogenic factors, for example caused by a de novo variant with large effect and polygenic factors, which is attributable to the cumulative effect of multiple common variants, each having a small effect^2^. In the past 20 years, there has been tremendous progress in identifying genes robustly associated with autism^3,4^ and more widely with neurodevelopmental disorders (NDDs)^5–7^, including cognitive impairment, delayed developmental milestones and epilepsy^8,9^.

Little is known about the prevalence of rare LoF variants within these genes in individuals without a diagnosis of autism. Nor do we understand the inter-individual phenotypic variability of carriers beyond the autism diagnosis^10,11^. In this study, we analyzed whole-exome sequencing (WES) data from four studies, for a total of 226,649 individuals of genetically inferred European ancestries (Supplementary Fig. 1 and Methods); 13,091 individuals diagnosed with autism, recruited in the Simons Simplex Collection (SSC), the Simons Powering Autism Research for Knowledge (SPARK) and the Lundbeck Foundation Initiative for Integrative Psychiatric Research (iPSYCH) projects, independently from co-occurring cognitive impairment or other NDDs (henceforth, individuals with autism), 19,488 first-degree relatives of individuals with autism from the SSC and SPARK projects and 194,070 individuals identified from unselected population samples of the iPSYCH and UK Biobank projects (Supplementary Fig. 2 and Methods). We quantified the odds ratios (ORs) of rare LoF variants in individuals with autism versus individuals not diagnosed with an NDD (henceforth, undiagnosed individuals) in genes previously associated with autism. We then compared the phenotypic profile of LoF carriers to non-carriers among both diagnosed and undiagnosed individuals. We show that rare LoF variants are associated with sub-diagnostic effects in individuals with autism and may also be associated with, on average, a small but significant effect on cognitive performance and socioeconomic status among unselected population individuals.

## Results

### Gene-level estimate of the odds ratio for autism

**Extended Data Fig. 1:**
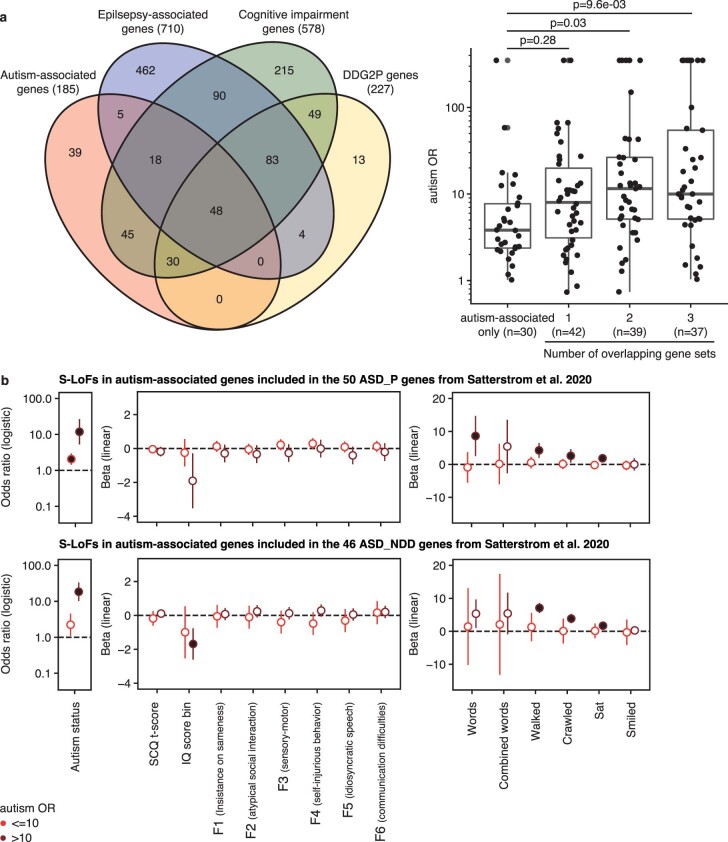
Effect of S-LoFs in genes associated to neurodevelopmental disorders in autistic individuals. (**a**) Overlap between the autism-associated genes and lists of genes associated to cognitive impairment, epilepsy or neurodevelopmental disorders, cataloged in Leblond et al. Mol Cell Neuro 2021 and updated in-house in March 2022 (left, available at https://genetrek.pasteur.fr/). The distribution of autism OR of genes overlapping with an increasing number of gene sets is shown (right), along with p values from two-sided Mann–Whitney U-tests, corrected for multiple testing using the Bonferroni method. The number of genes in each category is shown. Box plots representing minimum, first quartile, median, third quartile, maximum values, with outliers defined as first quartile minus 1.5 times the interquartile range and third quartile plus 1.5 times the interquartile range. (**b**) Multivariable regressions restricted to genes annotated as ‘ASD_P’ or ‘ASD_NDD’ in Satterstrom et al. Cell 2020. Legend as in Fig. 4a.

First, we listed a set of 185 autosomal genes with dominant mode of inheritance that are more frequently mutated in individuals with autism than in undiagnosed individuals (Supplementary Table 1 and Methods)^8^. We refer to these genes as ‘autism-associated genes’ despite no evidence linking these genes specifically to autism compared to other neurodevelopmental conditions (Extended Data Fig. 1)^5,6,12^ and recent evidence for association of rare de novo variants in autism-associated genes with autism and co-occurring cognitive impairment^7^. In addition, we analyzed 2,492 genes not considered as autism-associated genes, but with evidence for intolerance to LoF variants in reference populations (hereafter referred to as ‘constrained genes’; Supplementary Table 1 and Methods)^13^.

**Fig. 1:**
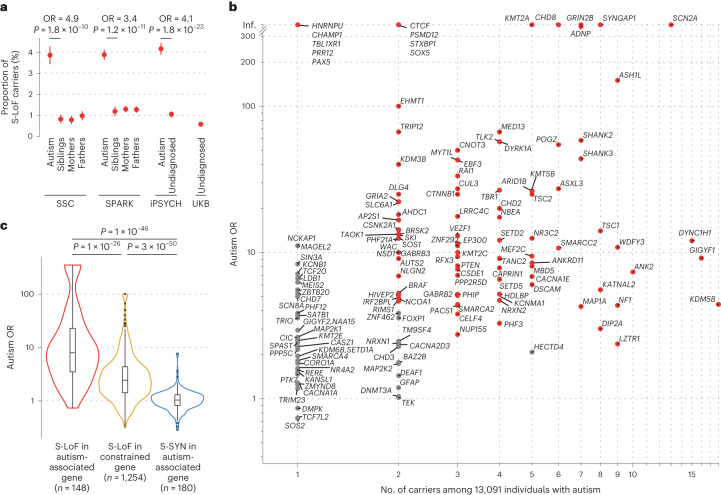
Gene-level autism odds ratio for rare variants in autism-associated and constrained genes. **a**, Proportion of individuals carrying high-confidence rare S-LoFs in autism-associated genes in each sample, stratified by status and family relationship. Error bars correspond to standard errors of the proportions. ORs and *P* values from two-sided Fisher exact tests comparing children with autism and their siblings in SSC and SPARK samples and individuals with autism and undiagnosed individuals in the iPSYCH sample. *P* values corrected for multiple testing using Bonferroni method for each variant type and gene set (SSC, *n* = 2,041 individuals with autism, 1,944 siblings, 2,041 mothers and 2,041 fathers; SPARK, *n* = 6,239 individuals with autism, 2,344 siblings, 5,559 mothers and 5,559 fathers; iPSYCH, *n* = 4,811 individuals with autism, 5,214 undiagnosed individuals; UK Biobank (UKB), *n* = 188,856 undiagnosed individuals). **b**, Number of S-LoF carriers among individuals with autism and autism OR, which is the enrichment of S-LoFs among individuals with autism compared to undiagnosed individuals (based on 100 sub-samplings of undiagnosed individuals to match the number of individuals with autism; Methods). Genes with autism ORs significantly higher than expected by chance (empirical test based on 10,000 bootstraps; Methods) are shown in red, others in gray. **c**, Distribution of gene-level autism OR of S-LoFs in autism-associated genes, S-LoFs in constrained genes and S-SYNs in autism-associated genes. Box plots representing minimum, first quartile, median, third quartile and maximum values, with outliers defined as first quartile minus 1.5 × interquartile range and third quartile plus 1.5 × interquartile range. *P* values are from two-sided Mann–Whitney *U*-tests.

**Extended Data Fig. 2:**
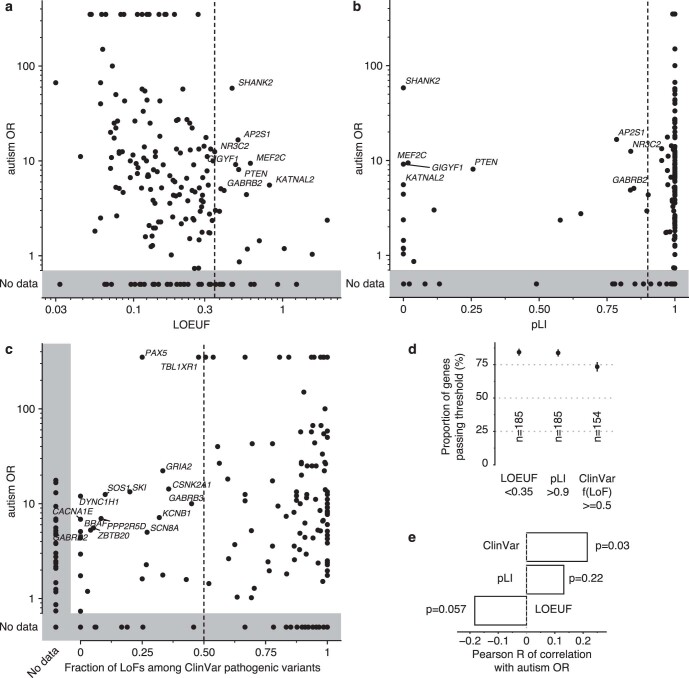
Comparison of autism OR with LoF deleteriousness scores from gnomAD and ClinVar pathogenic variants for autism-associated genes. The suggested LOEUF threshold of 0.35 (a), pLI threshold of 0.9 (b) and 50% of pathogenic variants that are LoF versus missense variants in ClinVar (c) are shown. (d) Fraction of autism-associated genes passing the thresholds for each metric. Error bars correspond to standard errors of the proportions. (e) Two-sided Pearson correlation coefficients and p values when comparing autism OR, pLI scores, LOEUF scores and fraction of LoFs among ClinVar pathogenic variants. P values were corrected for multiple testing using the FDR method. The ClinVar database (https://www.ncbi.nlm.nih.gov/clinvar/) was downloaded in July 2022, variants annotated as ‘pathogenic’ were extracted and separated between LoF (‘nonsense’, ‘splice_acceptor_variant’, ‘frameshift_variant’, ‘splice_donor_variant’, ‘stop_lost’) and missense variants based on the consequence field.

Second, we identified high-confidence rare LoF variants (frequency <1% in each study) that were absent from the reference European population in the Genome Aggregation Database (gnomAD; https://gnomad.broadinstitute.org/)^13^. We focused this study on LoF variants because 80% of known autism-associated genes are considered as intolerant to LoF variants and 73% are predominantly reported with LoF pathogenic variants in ClinVar (Extended Data Fig. 2)^13,14^. Because the impact of a LoF variant might depend on its location in the coding region^13,15^, we further selected a subset of these LoF variants that fell in an exon retained in >10% of the brain transcripts of the corresponding gene and truncated >10% the encoded protein (Methods). We refer to this subset as stringent LoFs (S-LoFs). We observed S-LoFs in autism-associated genes in 4% of individuals with autism (*n* = 523, 95% confidence interval (CI) 3.66–4.33%), 1.13% of their siblings and parents (*n* = 223, 95% CI 0.99–1.29%) and 0.58% of individuals from UK Biobank (*n* = 1,090, 95% CI 0.54–0.61%; Fig. 1a). We also observed that 36% of the S-LoFs in autism-associated genes identified among undiagnosed individuals fall within the same exons as those identified among individuals with autism (Supplementary Fig. 3), suggesting that these variants should have very similar consequences on the encoded protein^16^.

**Extended Data Fig. 3:**
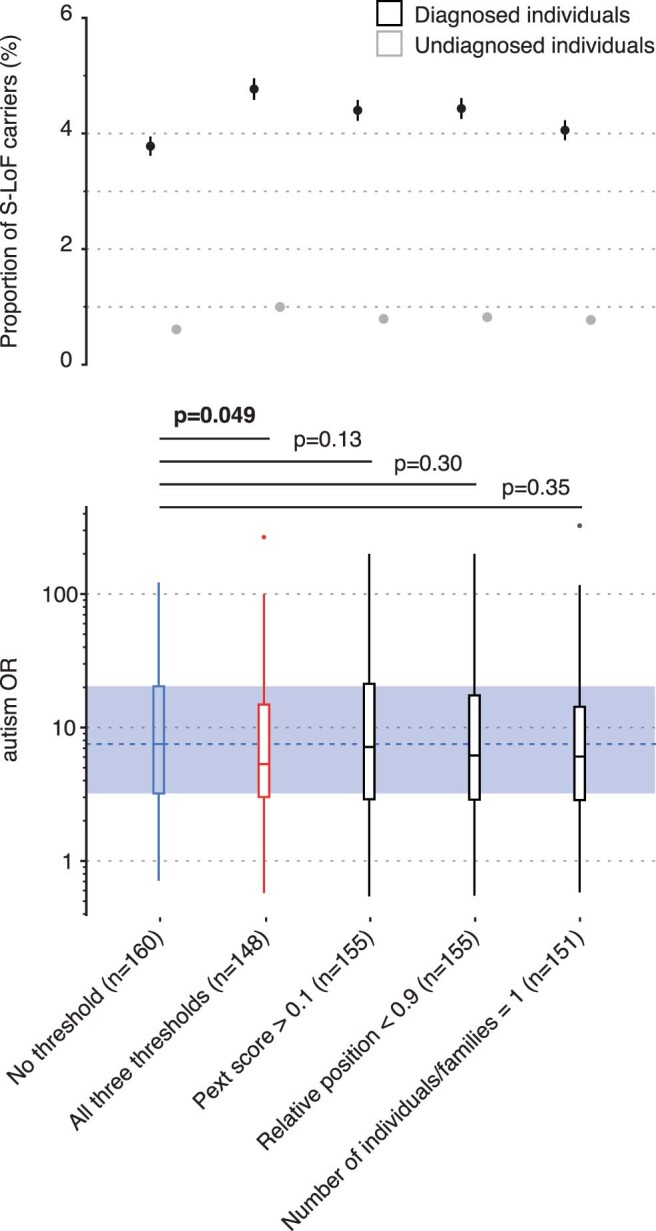
Gene-level autism OR as a function of number of carrying individuals or families, average pext in brain tissues and relative position in encoded protein. Proportion of individuals carrying S-LoFs stratified by autism status (top), and corresponding gene-level autism ORs (bottom) as a function of thresholds in pext score, relative position on encoded protein and number of individuals or families. The fraction of undiagnosed individuals carrying S-LoFs corresponds to the average fraction of individuals in the 100 sub-sampling (Methods). Error bars correspond to standard errors of the proportions. The thresholds correspond to S-LoFs that were present in more than 10% of the brain-expressed transcripts, truncating more than 10% of the encoded protein, that is not in the last 10% of the protein sequence, and/or found in only one family or individual. The number of genes for which we find at least one diagnosed individual carrying a variant is indicated. Box plots representing minimum, first quartile, median, third quartile, maximum values, with outliers defined as first quartile minus 1.5 times the interquartile range and third quartile plus 1.5 times the interquartile range. P values from two-sided Mann–Whitney U-tests.

We then estimated for each gene the OR for autism (autism OR) of S-LoFs (Fig. 1b), which is the enrichment of S-LoFs among individuals with autism versus undiagnosed individuals, adjusting for the large difference in sample size between individuals with autism and undiagnosed individuals using a sub-sampling procedure (Extended Data Fig. 3 and Methods). Prevalence, autism OR and aggregated variant data can be visualized and downloaded at https://genetrek.pasteur.fr/ ref. ^12^. Several autism-associated genes such as *SCN2A*, *ASH1L* and *ANK2* had the highest number of S-LoFs identified among individuals with autism (Fig. 1b), but they displayed distinct frequencies of S-LoFs among undiagnosed individuals, therefore displaying distinct autism ORs (for example, *SCN2A* = Inf.; *ASH1L* = 150.1; and *ANK2* = 7.4). *SCN2A* was among 14 autism-associated genes (Supplementary Table 1) such as *CHD8*, *GRIN2B* and *SYNGAP1* for which all variants identified in individuals with autism were found de novo^17^ and for which no carriers of S-LoFs were identified among the 213,558 undiagnosed individuals. In contrast, for 134 autism-associated genes, including *ASH1L*, *ANK2* and *SHANK3* (Supplementary Fig. 3), we could identify at least one carrier of an S-LoF among the undiagnosed individuals, suggesting lower effect sizes on autism diagnosis (Fig. 1b and Supplementary Table 1). We observed that four genes (*AP2S1*, *GIGYF1*, *PTEN* and *SHANK2*) displayed an autism OR > 8, whereas they were not classified as LoF-intolerant based on variant frequency in the general population (Supplementary Table 1)^13^, supporting caution in applying specific cutoffs for LoF intolerance metrics^18^. We also observed that autism-associated genes also previously reported as associated with cognitive impairment, epilepsy or developmental disorders had higher autism ORs than those that were not (Extended Data Fig. 1)^12^. Altogether our results indicate that an exhaustive investigation of less penetrant variations is warranted to better understand the association of genes with autism and more generally with NDDs^19,20^.

**Extended Data Fig. 4:**
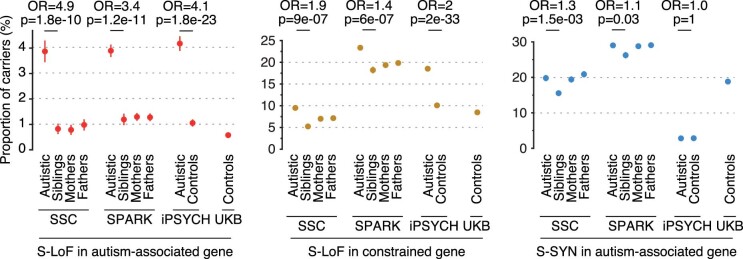
Proportion of individuals carrying S-LoFs in autism-associated genes, S-LoFs in constrained genes or S-SYNs in autism-associated genes. Proportions are shown in each sample, stratified by status and family relationship. Odds ratios and p values from two-sided Fisher exact tests. Error bars correspond to standard errors of the proportions. P values corrected for multiple testing using Bonferroni method for each variant type and gene set. SSC: Simons Simplex Collection (n = 2,041 individuals with autism, 1,944 siblings, 2,041 mothers and 2,041 fathers), SPARK: Simons Powering Autism Research for Knowledge (n = 6,239 individuals with autism, 2,344 siblings, 5,559 mothers and 5,559 fathers), iPSYCH: The Lundbeck Foundation Initiative for Integrative Psychiatric Research (n = 4,811 individuals with autism, 5,214 undiagnosed individuals), UKB: UK Biobank (n = 188,856 undiagnosed individuals).

To compare the effect of S-LoFs in autism-associated genes with other types of variants and sets of genes, we subsequently measured the autism OR of synonymous variants in autism-associated genes (S-SYNs; using similar filters as S-LoFs based on exon usage in brain, position on encoded protein and frequency) and of S-LoFs in 2,492 constrained genes (Extended Data Fig. 4 and Supplementary Table 1). As expected, S-LoFs in autism-associated genes displayed higher autism ORs compared to S-LoFs in constrained genes (nominal *P* = 1 × 10^−26^) and S-SYNs in autism-associated genes (nominal *P* = 1 × 10^−46^; two-sided Mann–Whitney *U*-test) (Fig. 1c). Notably, some constrained genes such as *AP2M1* and *CACNG2*, reported in individuals with cognitive impairment, displayed autism ORs >10 without being included in the lists of autism-associated genes (for example SFARI and SPARK genes).

**Fig. 2:**
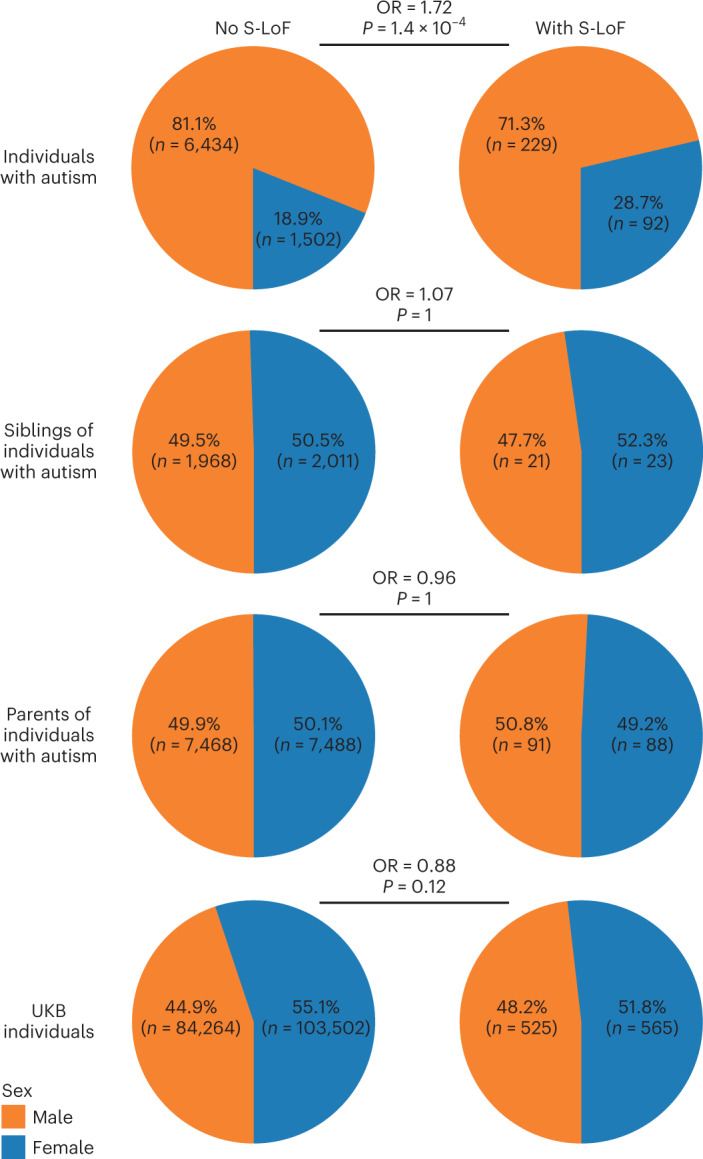
Sex ratio among carriers and non-carriers of S-LoFs in autism-associated genes. Pie charts of the fraction of male and female individuals among non-carriers and carriers of S-LoFs in autism-associated genes, stratified by status and family relationship. ORs for enrichment of S-LoFs among female over male individuals and corresponding *P* values from two-sided Fisher exact tests. *P* values were corrected for multiple testing using the Bonferroni method.

We found a significant enrichment of female individuals with autism carrying S-LoFs in autism-associated genes compared to male individuals with autism (OR 1.72, *P* = 1.4 × 10^−4^, Fisher exact test), as previously reported^21,22^, but no difference was found among undiagnosed siblings, parents and individuals from the unselected population (Fig. 2).

### Relationship between biological functions and autism OR

**Fig. 3:**
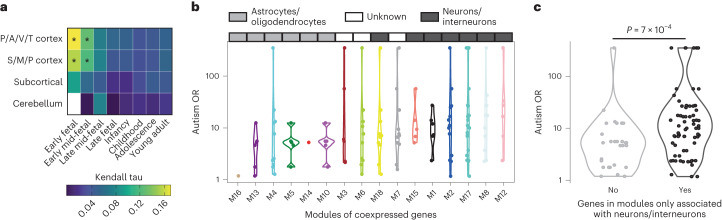
Relationship between gene expression profile and autism OR. **a**, Correlation between autism OR and gene expression in distinct brain regions and developmental periods for 130 genes for which at least one variant was identified among individuals with autism (expression data for early fetal cerebellum were not available; Methods). Cortical regions were grouped as follows: posterior inferior parietal cortex, primary auditory cortex, primary visual cortex, superior temporal cortex and inferior temporal cortex (P/A/V/T cortex); primary somatosensory cortex, primary motor cortex, orbital prefrontal cortex, dorsolateral prefrontal cortex, medial prefrontal cortex and ventrolateral prefrontal cortex (S/M/P cortex). Correlations and *P* values measured by two-sided Kendall correlation tests between autism OR and gene expression (*nominal *P* < 0.05; Supplementary Table 2 shows exact values). **b**, Distribution of autism OR of autism-associated genes in different brain coexpression modules. Brain coexpression modules were extracted from Voineagu et al.^24^. Modules are ordered according to average autism OR of corresponding genes. Modules were mapped to cell types in the original study. **c**, Distribution of autism OR of autism-associated genes found exclusively in modules associated with neuron or interneuron cell types and those in modules associated both with neuron/interneuron and other cell types. *P* values are from two-sided Mann–Whitney *U*-tests. For **a**–**c**, we set infinite autism OR values to the highest measurable autism OR in the corresponding gene set.

To investigate the relationship between biological functions and the autism OR, we studied the expression level of autism-associated genes in four different human brain regions and at eight different developmental periods. We found that the autism OR tended to be positively correlated with gene expression in early fetal and mid-fetal periods of cortex development (nominal *P* < 0.05 in auditory, visual, parietal and temporal cortex at the early fetal and mid-fetal periods, Fig. 3a, Supplementary Table 2 and Methods)^23^.

**Extended Data Fig. 5:**
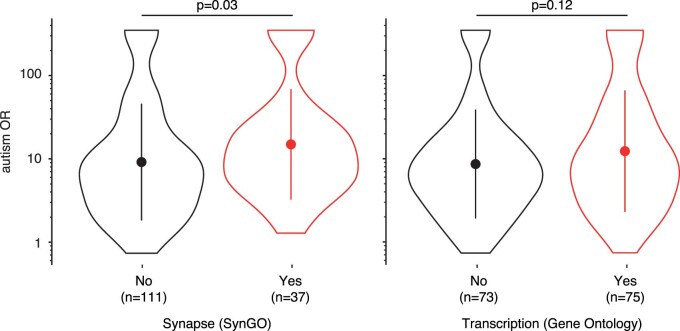
Biological pathways associated to high autism OR. Distribution of autism OR for genes encoding synaptic and transcription proteins compared to autism OR of genes not encoding such proteins. Dots correspond to mean values and error bars to standard deviations. P values from two-sided Mann–Whitney U-tests.

We also investigated the autism OR of genes in modules of coexpressed genes previously reported as significantly different between autism and control brains^24^. We observed that the modules enriched in neuronal markers included the genes with the highest autism OR compared to modules enriched for astrocyte and oligodendrocyte markers (Fig. 3b,c and Methods), with the highest average autism OR being observed for the module showing the highest correlation with autism diagnosis (M12) associated with synaptic functions. Using gene annotation for the 185 autism-associated genes, we also observed that genes encoding proteins associated with synapse function/architecture tended to display higher autism ORs compared to genes not encoding synaptic proteins (nominal *P* = 0.03; Extended Data Fig. 5 and Supplementary Table 3).

### Phenotypic effects of variants among individuals with autism

**Fig. 4:**
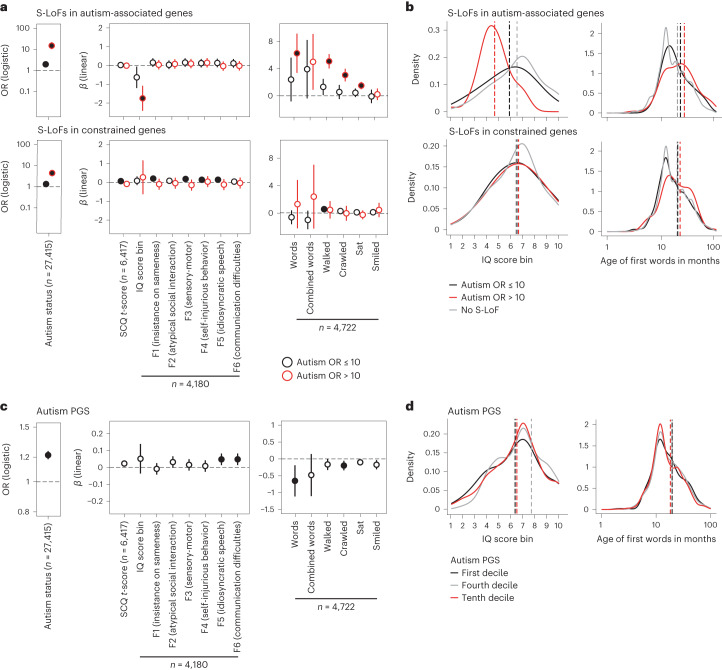
Phenotypic effects of rare variants in autism-associated and constrained genes among diagnosed individuals. **a**, OR (for logistic regressions) and *β* values (for linear regressions) associated with variant presence from multivariable regression analyses of autism diagnosis, SCQ *t*-score, IQ score bin, autism factors and developmental milestones, stratified by gene type and autism OR of genes carrying the variants (Methods). Regressions performed on individuals from the SSC and SPARK samples. To correct for the biased sex ratio among individuals with autism, with approximately one female to four males, sex was added as a covariate. Error bars correspond to 95% CI. *P* values associated with each *β* value were corrected for multiple testing using the false discovery rate (FDR) method (full circles correspond to corrected *P* < 0.05). The number of individuals with available data is shown for each regression. For age at developmental milestones, age is given in months and higher values indicate higher age. **b**, Distribution of trait values for IQ score bin and age of first words for carriers and non-carriers of S-LoFs. Vertical lines indicate average values. **c**, *β* values associated with autism PGS from multivariable regression analyses of autism diagnosis, SCQ *t*-score, IQ score bin, autism factors and developmental milestones. *β* values associated with autism PGS correspond to regression analyses with S-LoFs in constrained genes with autism OR > 10 considered as covariates (Supplementary Tables 4 and 5 show complete results). Regressions performed on individuals from the SSC and SPARK samples. To correct for the biased sex ratio among individuals with autism, with approximately one girl to for boys, sex was added as a covariate. Error bars correspond to 95% CI. *P* values associated with each *β* value were corrected for multiple testing using the FDR method (full circles correspond to corrected *P* < 0.05). The number of individuals with available data is shown for each regression. For age at developmental milestones, age is given in months and higher values indicate higher age. **d**, Distribution of trait values for IQ score bin and age of first words for individuals in the first, fourth and tenth decile of the autism PGS distribution. Vertical lines indicate average values.

**Extended Data Fig. 6:**
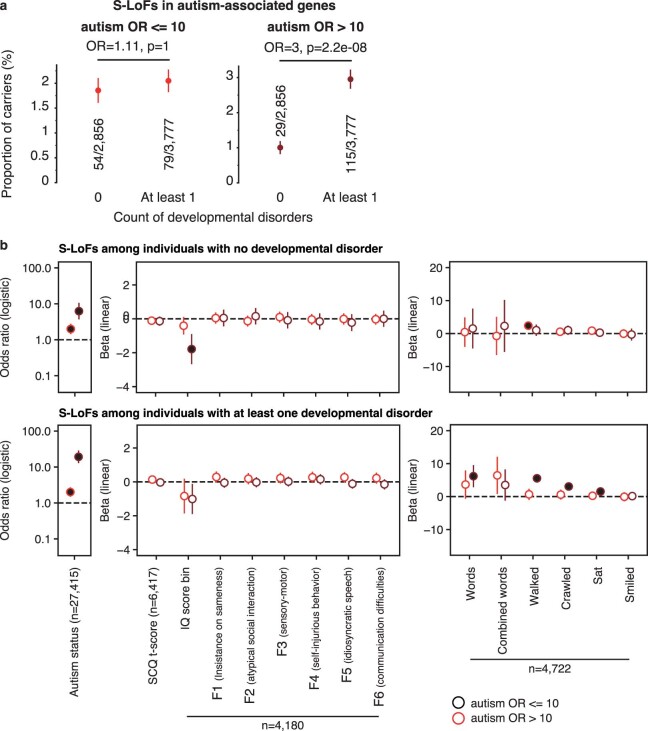
Effect of S-LoFs among syndromic and non-syndromic autistic individuals. (**a**) Proportion of individuals carrying S-LoFs among individuals with autism that present no developmental disorder (n = 2,856 individuals) or at least one developmental disorder (n = 3,777 individuals), for S-LoFs in autism-associated genes with autism OR ≤ 10 or autism OR > 10. Odds ratio and p values from two-sided Fisher exact tests. Error bars correspond to 95% confidence intervals. P values corrected for multiple testing using the Bonferroni method. The number of carriers and non-carriers are shown. (**b**) Multivariable regressions among individuals without developmental disabilities or with at least one developmental disorder. Error bars correspond to 95% confidence intervals. Legend as in Fig. 4a.

**Extended Data Fig. 7:**
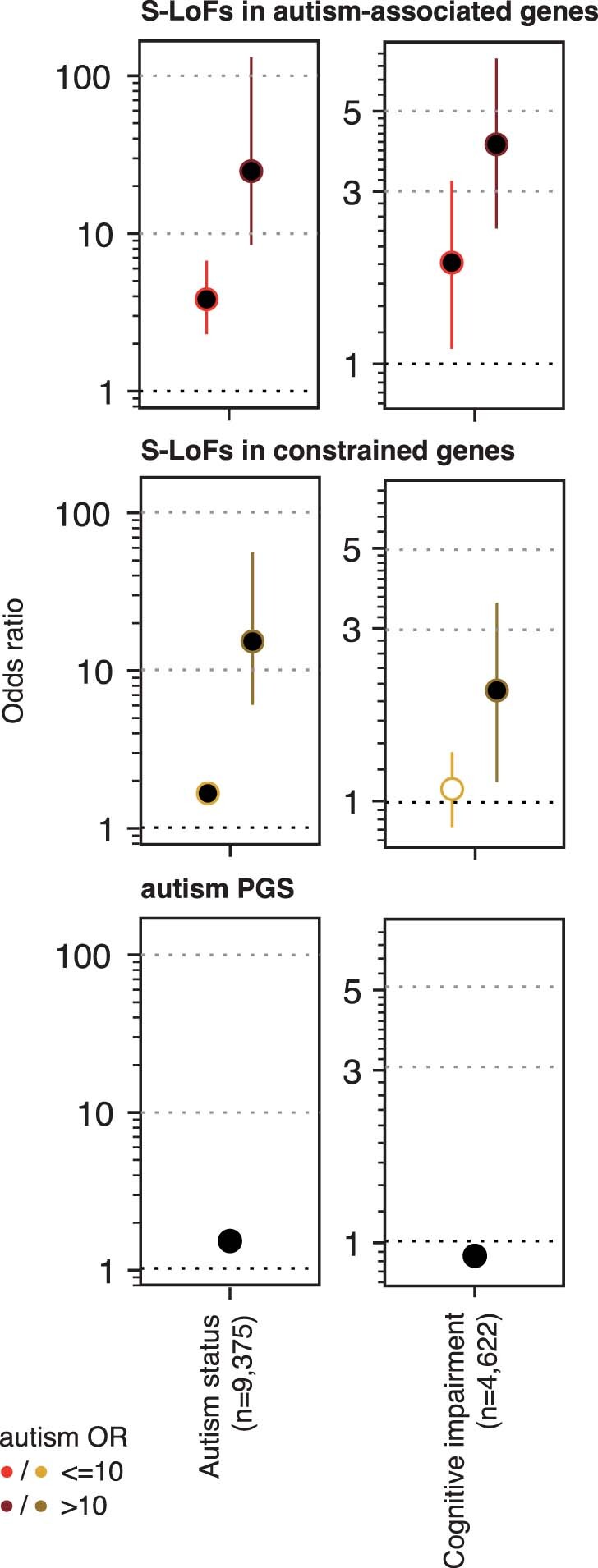
Regression analysis for the effect of S-LoFs and autism PGS on autism status and cognitive impairment in the iPSYCH sample. Odds ratio associated to variant presence and autism PGS from multivariable regression analyses of autism status and cognitive impairment (Methods). The odds ratio associated to autism PGS when S-LoFs in constrained genes with autism OR > 10 are considered in the regression analysis are shown. Error bars correspond to 95% confidence interval. P values associated with each beta value were corrected for multiple testing using the FDR method (full circles indicate corrected p < 0.05). The number of individuals with available data is shown.

Besides rare variants with large effect, common variants associated with autism have been identified through genome-wide association studies (GWAS) and can be aggregated to calculate a polygenic score (PGS) for autism for each individual (Supplementary Fig. 4 and Methods)^2,25,26^. Using logistic regression models, we estimated the independent and interaction effects on autism diagnosis due to the S-LoFs and the autism PGS for 27,212 individuals, including 8,089 individuals with autism and 19,123 relatives from the SSC and SPARK cohorts. We distinguished S-LoFs in genes below and above a threshold of autism OR of 10 to quantify their differential effect on the autism diagnosis. We note here that this approach allows to estimate a general association between genetic variants and phenotypic outcomes and not a direct causal relationship. In a subset of 6,910 individuals with available phenotypic data, S-LoFs in genes with autism OR > 10 were enriched among individuals with at least one reported developmental disorder compared to those without a reported developmental disorder (Extended Data Fig. 6). Associations of S-LoFs, autism PGS and sex with autism status were all significant (Fig. 4a and Supplementary Tables 4 and 5). The effect size of S-LoFs with autism status was 1.8–2.3-times higher for S-LoFs in autism-associated genes than in constrained genes and 3.4–13.6-times higher for S-LoFs in autism-associated genes than for an increase of one standard deviation of the autism PGS (Fig. 4a,c). We replicated these results in an independent analysis of the iPSYCH sample (Extended Data Fig. 7, Supplementary Table 4 and Methods).

We performed additional multivariable regression analyses to investigate the effect of S-LoFs and autism PGS on several traits, including age at developmental milestones, the social and communication questionnaire (SCQ) *t*-score, the intelligence quotient (IQ) score bins and six main autism-related factors previously described^7^ (F1, insistence on sameness; F2, atypical social interaction at age 5 years; F3, atypical sensory-motor behavior; F4, self-injurious behavior; F5, idiosyncratic repetitive speech and behavior; and F6, difficulties in communication) (Fig. 4a and Supplementary Tables 4 and 5). No significant association of S-LoFs with SCQ *t*-score or autism-related factors were observed; however, we observed a significant negative association of S-LoFs in autism-associated genes with IQ score bins, replicated in the independent iPSYCH sample (Extended Data Fig. 7) and a positive association with age at developmental milestones, supporting the previously reported associations of de novo variants with IQ and developmental milestones among children with autism^7,27^. These effects were (1) higher for genes with autism OR > 10 (Fig. 4a,b); (2) observed both among individuals with autism with and without developmental disorders (Extended Data Fig. 6); and (3) both among genes proposed to be associated predominantly with neurodevelopmental disorders or with autism^8^ (Extended Data Fig. 1 and Supplementary Table 4). Notably, S-LoFs in constrained genes were significantly associated with SCQ *t*-score and autism factors (F1, F3, F4 and F5) but not with IQ score bins and developmental milestones, with the exception of age of walking (Fig. 4a). The autism PGS was associated with factors related to difficulties in speech and communication (F5 and F6), suggesting an effect of the common variants on communication skills and repetitive speech/behaviors in individuals with autism (Fig. 4c,d). Finally, we did not observe interaction between S-LoF and autism PGS, suggesting that currently in this setting, the effects of rare and common variants associated with autism-related traits are mostly independent^25^.

### Phenotypic effects of rare variants among undiagnosed individuals

**Fig. 5:**
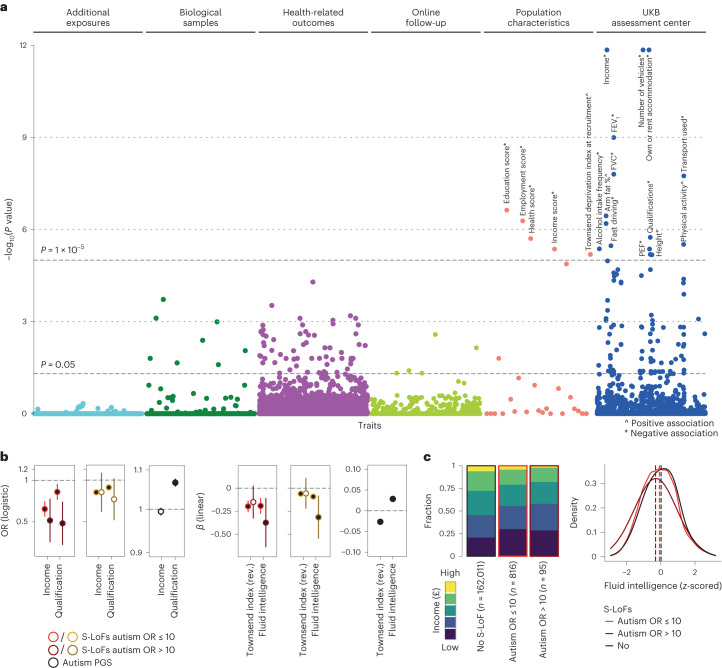
Phenotypic effects of rare variants in autism-associated and constrained genes among undiagnosed individuals. **a**, Phenome-wide association study of the effect of S-LoFs in autism-associated genes using the PHESANT software on 18,224 traits from the UK Biobank (Methods). *P* values were corrected for multiple testing using the FDR method and shown for all tested phenotypes (complete results shown in Supplementary Table 6). Traits were classified according to the broad category defined in the UK Biobank database. FVC, forced vital capacity; FEV_1_, forced expiratory volume in 1 s; PEF, peak expiratory flow. **b**, OR (logistic regressions) and standardized *β* values (linear regressions) associated with variant presence and autism PGS from multivariable regression analyses of socioeconomic traits and fluid intelligence, stratified by gene type and autism OR of genes carrying the variants (Methods). The Townsend index measures were reversed so that higher material deprivation was indicated with a negative sign. The *β* values associated with autism PGS when S-LoFs in constrained genes with autism OR > 10 are considered in the regression analysis are shown (Supplementary Tables 4 and 5 show complete results). Error bars correspond to 95% CI. *P* values associated with each *β* value were corrected for multiple testing using the FDR method (full circles correspond to corrected *P* < 0.05). The number of individuals used in the regression analyses was as follows: fluid intelligence, *n* = 112,614; income, *n* = 162,968; qualification, *n* = 156,483; and Townsend deprivation index, *n* = 188,630. **c**, Distribution of incomes and fluid intelligence scores are shown for carriers and non-carriers of S-LoFs in autism-associated genes among undiagnosed UK Biobank individuals.

**Extended Data Fig. 8:**
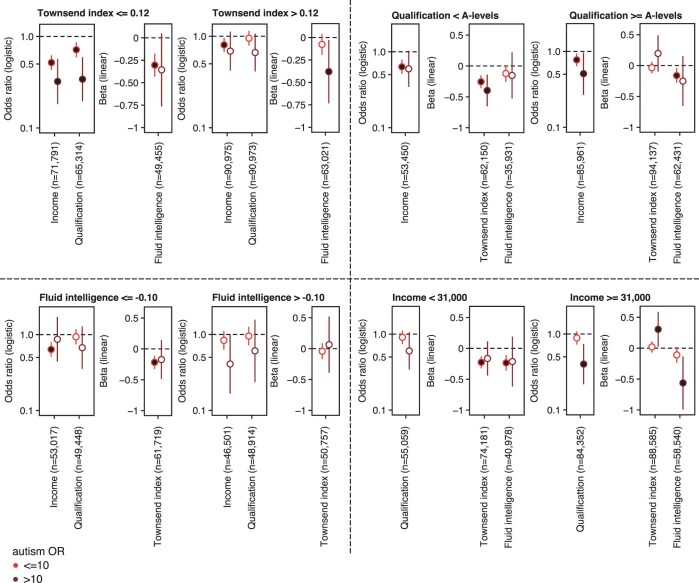
Regression results for socioeconomic and cognitive traits in different socioeconomic and cognitive strata. Odds ratio (logistic regressions) and standardized beta values (linear regressions) associated to variant presence and autism PGS from multivariable regression analyses of socioeconomic traits and fluid intelligence, stratified by gene type and autism OR of genes carrying the variants, alternatively focusing on individuals within low and high range of values for each feature (Methods). For the Townsend index and fluid intelligence, the median of the distribution of values among S-LoF carriers was used to split the dataset (respectively z-scored reversed Townsend index of 0. 12092671 and fluid intelligence score of −0.10951938). For income, we chose to split individuals below and above £31,000, and for qualification below and above A levels or equivalent. This procedure allowed to split individual carrying S-LoFs into two partitions of approximately the same size. Error bars correspond to 95% confidence interval. Legend as in Fig. 5b.

We subsequently explored whether, among participants of the UK Biobank without a recorded diagnosis of autism, carriers of S-LoFs displayed differences in any phenotypic trait compared to non-carriers. We interrogated 18,224 traits in a phenome-wide association study and found that the most significant associations were observed for unemployment, income, qualification and Townsend deprivation index, which is a measure of material deprivation within a population (corrected *P* < 1 × 10^−5^; Fig. 5a, Supplementary Table 6 and Methods). Multivariable regression analysis on fluid intelligence scores, which is a simple unweighted sum of the number of correct answers given to the 13 fluid intelligence questions (Methods), qualification levels, income and material deprivation estimated by the Townsend deprivation index (for example, unemployment and non-home ownership) (Supplementary Table 5), showed that individuals carrying S-LoFs in autism-associated genes displayed on average lower fluid intelligence (estimated *β* = −0.19 and −0.37 for S-LoFs in genes with autism OR ≤ 10 and >10, respectively), qualification (estimated OR = 0.82 and 0.49), income (estimated OR = 0.62 and 0.51) and higher material deprivation (estimated *β* = −0.2 and −0.15 for reversed Townsend index) compared to non-carriers (Fig. 5b,c). These associations were stronger for S-LoFs in autism-associated genes than in constrained genes. We further investigated the effect of S-LoFs within more homogeneous subgroups based on their cognitive and socioeconomical scores and observed that the highest effect sizes of S-LoFs were found for the subgroups of individuals with lower scores of fluid intelligence, income, qualification and higher scores of the Townsend deprivation index (Extended Data Fig. 8). Notably, in contrast to the impact of S-LoFs, the autism PGS was positively associated with fluid intelligence and qualification level; however, as for S-LoFs, the autism PGS was also associated with increased level of the Townsend deprivation index (Fig. 5b). Altogether our results on a large sample of individuals with autism and undiagnosed individuals indicate that S-LoFs mostly affect the cognitive skills of individuals rather than their socio-communication abilities, as previously reported for large copy-number variants or de novo single-nucleotide variants^7,28–31^.

**Fig. 6:**
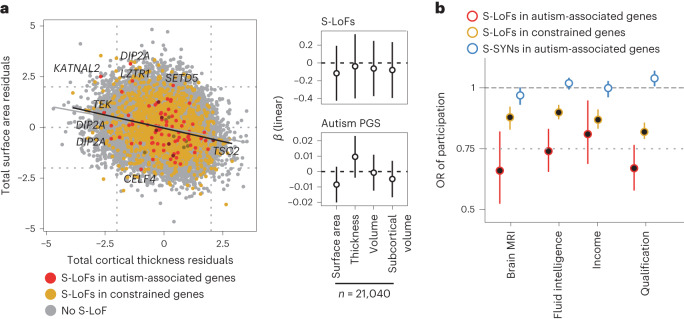
Effect of genetic variants on brain anatomy and participation to questionnaires in the UK Biobank. **a**, Distribution of total cortical surface area and thickness for carriers and non-carriers of S-LoFs in autism-associated or constrained genes (left). The values shown were corrected for age, sex and scanning site. Gene symbols are indicated for individuals with surface area or thickness values over 2 standard deviations from the mean (*z*-scored values <−2 or >2) and carrying S-LoFs in autism-associated genes. Standardized *β* values associated with variant presence and autism PGS from multivariable regression analyses of brain anatomy among UK Biobank undiagnosed individuals (right). Error bars correspond to 95% CI. Total values for cortical thickness, surface area and volume were measured as the sum of all 68 regions (Methods). S-LoFs in autism-associated and constrained genes were grouped to increase sample size (Supplementary Table 7 shows complete results). *P* values were corrected for multiple testing using the FDR method. **b**, OR of participation when carrying a variant among UK Biobank undiagnosed individuals (*n* = 188,856 individuals) for S-LoFs in autism-associated and constrained genes and for S-SYNs in autism-associated genes. Error bars correspond to 95% CI. *P* values were corrected for multiple testing using the FDR method for each gene set and variant type independently (full circles indicate corrected *P* values < 0.05).

**Extended Data Fig. 9:**
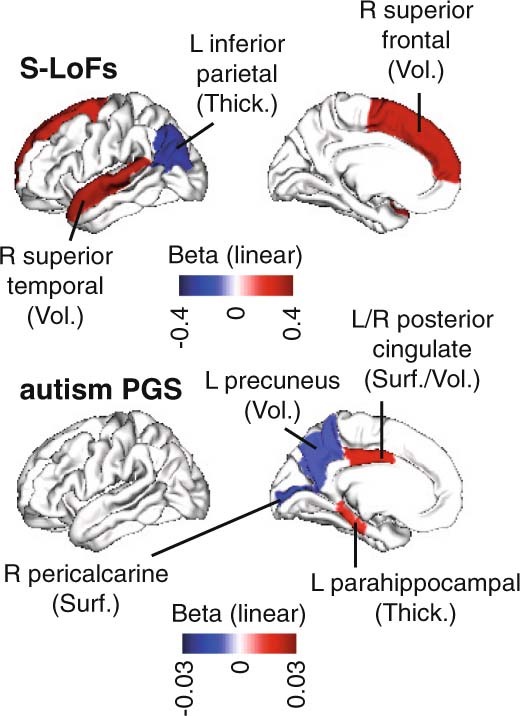
Brain maps showing the standardized beta coefficients associated to variant presence and autism PGS. Standardized beta coefficients associated to variant presence and autism PGS from multivariable linear regression analyses of brain sub-regions. P values were corrected for multiple testing using the FDR method, and only sub-regions with corrected p values below 0.05 are shown. Beta coefficients from the two hemisphere and from the three metrics were merged, and corresponding hemispheres and metrics for each sub-region are displayed.

Several autism-associated variants have been shown to modify brain structure^32–34^ and we finally questioned whether S-LoFs or the autism PGS had an impact on brain anatomy using magnetic resonance imaging (MRI) data from 21,040 UK Biobank individuals. To increase our prediction power, we grouped the 1,675 carriers of S-LoFs in autism-associated or in constrained genes and tested whether carriers of S-LoFs displayed differences in global and regional cortical volume, thickness and surface area, as well as global and regional subcortical volume, using multivariable linear regression analyses (Supplementary Table 7 and Methods). The age, sex and scanning site of individuals were added as covariates to account for their effect on the variation in brain structure. We observed that neither S-LoFs nor autism PGS was associated with differences in distribution of global cortical or subcortical metrics (Fig. 6a) and that S-LoFs carriers did not display higher deviation in these metrics than non-carriers (Supplementary Table 7). We found significant associations of S-LoFs and autism PGS with some specific brain regions (Extended Data Fig. 9), which seemed largely independent from environmental factors such as early-life trauma, which were previously shown to contribute to brain anatomy differences^35^ (Supplementary Fig. 5 and Supplementary Table 7). Notably, partitions of the autism PGS based on specific gene sets were associated with anatomical metrics of different brain regions (Supplementary Fig. 6). The investigation of the genetic and environmental context that contribute to such brain structure differences would, however, require larger sample sizes^36^.

UK Biobank individuals are not a perfectly accurate representation of the general population^37^ and participation bias has a genetic component^38,39^. We observed a significant negative effect of S-LoFs on response to questionnaires exploring qualification level, income and fluid intelligence (Fig. 6b, Supplementary Table 8 and Methods). This effect was higher for S-LoFs in autism-associated genes than for constrained genes and was absent for S-SYNs in autism-associated genes. Participation in brain MRI scanning showed the same trend, suggesting that the imaging subsample also presents a participation bias^40^. These results provide additional support that the UK Biobank sample may suffer from a ‘healthy volunteer bias’, which alters our ability to quantify the actual effect of genetic variants.

## Discussion

In summary, by systematically analyzing WES data of more than 13,000 individuals with autism and 210,000 undiagnosed individuals, we estimated the autism OR of rare LoF variants in 185 genes associated with autism. As expected, the genes with the highest autism ORs (for example *DYRK1A*, *GRIN2B*, *SCN2A* and *SYNGAP1*) were those repeatedly identified as affected by de novo variants in independent genetic studies of autism. The reasons why some individuals carrying the S-LoF will have a diagnosis of autism and some do not, probably depend on additional genetic, societal and environmental factors. In addition, the location of the variant in the encoded protein can be critical^41^. We found two undiagnosed individuals who carried S-LoFs impacting *SHANK3* (Supplementary Fig. 3), but these variants were identified in exons located in the 5′ region of the gene and affected the α-isoform of *SHANK3*, which was known to be associated with milder phenotypes^42^ compared to other isoforms^43^. Hence, in addition to a gene-level estimation, an exon or even site-specific estimation might be more accurate to assess the penetrance of the LoF variants^44^, but this level of accuracy will require even larger sample size cohorts.

In the unselected (or undiagnosed) population, we observed a correlation between carrying a S-LoFs and having lower income, qualification level and fluid intelligence and higher material deprivation (Fig. 5b, Supplementary Table 9 and Methods). This small effect on the socioeconomic status of the carriers is expected for LoF variants in genes known to be associated with cognitive impairment in individuals with autism (Fig. 4a,b)^7^. The underlying mechanisms linking the presence of genetic variants to the various social and health-related outcomes are complex and our findings do not represent causal relationships. For instance, these relationships could reflect generational effects (differences in expectations between individuals from different generations) or the fact that society does not provide adequate support to individuals with increased genetic likelihood for autism. Of note is the inverse relationship between autism PGS and fluid intelligence and income. Increasing autism PGS is associated with increase in fluid intelligence scores but reduced income, in stark contrast to the positive correlation observed between intelligence and income^45^. Although speculative, this could be indicative of the lack of social support that does not enable this group of individuals to flourish economically. The UK Biobank is also not entirely representative of the general population and the results warrant replication in an external cohort and additional research should be made to identify genetic, social and environmental resilience factors that influence how individuals with certain characteristics can flourish better.

**Extended Data Fig. 10:**
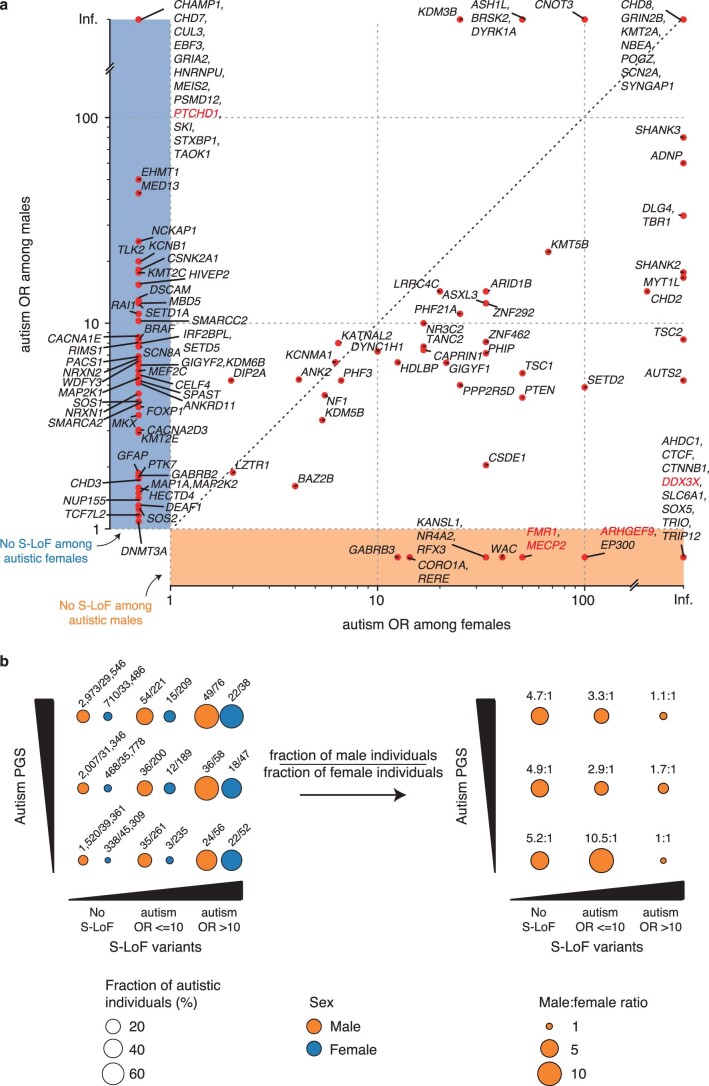
Autism OR among male and female individuals. (**a**) For each autism-associated gene, the autism OR among male individuals is compared to the autism OR among female individuals. Some genes were not found mutated among either male or female individuals with autism. The gene-level autism OR was measured using the sub-sampling procedure described in Methods, randomly selecting 1,596 and 6,683 individuals, that is the total number of female and male individuals with autism in the studied sample, for each autism status 100 times. For genes on the X chromosome (highlighted in red), we selected genes with dominant mode of inheritance for female individuals (for example MECP2), and we did not filter for inheritance mode for male individuals. (**b**) Fraction of individuals with autism (left) and male:female ratio (right) stratified by S-LoF presence and autism PGS. S-LoFs were divided between those identified in genes with autism OR below or above 10, and autism PGS was divided into terciles. For male/female ratios, the estimated numbers are shown.

Sex could be a factor modulating the penetrance of genetic variants. For some specific genes or pathways, penetrance of genetic variants could be different in males and females^1,11,46^. For example, inherited variants in autosomal genes such as *SHANK1* have been reported to be more frequently transmitted by mothers and lead to autism preferentially or exclusively in males^47^. In our study, we observed a significant enrichment of females with autism carrying S-LoFs in autism-associated genes compared to males with autism, as previously reported^21,22^. While our sample size was relatively large, it was not large enough to robustly investigate the gene-level autism OR of S-LoFs for males and females independently (Extended Data Fig. 10). We did not observe overall differences in sex ratio among non-autistic carriers of S-LoFs affecting autism-associated genes, as previously reported for parents of children with NDDs^48^ or for non-autistic siblings^8,46^. These results suggest that males and females are equally sensitive to S-LoFs in autism-associated genes. A potential explanation could be that S-LoFs are more prevalent genetic factors of autism in females because they may be less sensitive to lower loads of rare genetic variations and lower autism PGS compared to males (Extended Data Fig. 10)^7,49^.

The genetic background could also modulate the penetrance of LoFs as recently reported in carriers of the 22q11 deletion in schizophrenia^50^. In our study, we observed significant independent effects of S-LoFs and autism PGS on autism-related traits, but could not detect a significant interaction between them, suggesting these two genetic factors act independently on autism^25^. Interactive effects, however, are difficult to demonstrate and we might be underpowered to detect such interaction^25^, especially if the interplay between rare and common variants diverges from one gene to another. Integration of additional polygenic scores based on functional gene sets and for other traits (for example attention deficit hyperactivity disorder, IQ or educational years), as well as data related to expression levels (expression quantitative trait loci) in larger samples, is warranted to better understand the modifier effects of common variants on the phenotype of carriers^50–52^ and to enhance our understanding of the biological pathways associated with autism^26,53^. Epigenetic/environmental and stochastic factors might also modulate the penetrance of the genetic variants, but large-scale data to detect their impact are lacking so far^54^.

Finally, social environments also influence whether people with autistic traits receive a diagnosis and there is still progress to be made on a societal level to enable people with all different neurological and developmental diversities to thrive. For example, educational settings might not be always tailored to the needs of individuals with autistic traits, which could have important consequences on their chances later in life. Such confounding factors should be considered in future studies investigating the association of genetic variants with autistic and, more generally, neurodevelopmental traits.

To conclude, we show that LoF variants in autism-associated genes do not always result in a clinical diagnosis of autism in individuals but could influence the global functioning of the carriers as indicated by cognitive and socioeconomic metrics. Such fine-grained investigation of the effect of variants in autism-associated genes has important consequences for clinical counseling as they support a complex interplay between gene-level variations and clinical outcome^55,56^. Genetic variations might directly affect protein function, but there is a long developing process shaped by environmental and stochastic factors that will ultimately lead to socioeconomic and cognitive phenotypes. Future large-scale studies integrating environmental data and sub-diagnostic criteria should allow a better understanding of how some individuals can cope with the consequences of carrying such variations. Large-scale projects such as UK Biobank or the ‘All of us’ research program^57^ will enable the investigation of individuals with similar genetic variants, but with different outcomes. Such projects should contribute to a better understanding of both risk and resilience in a larger context taking into account developmental diversity and genetic, social and environmental factors.

## Methods

### Ethical approval

Informed consents from all individuals were obtained according to following ethics clearances. The SSC is a multisite effort gathering 12 recruitment sites and informed consents were obtained from all participants included in each site at the time of their initial enrollment and centralized by the Columbia University Institutional Review Board (IRB) under the protocol AAAC6306(M00Y17). All SPARK participants were recruited under a centralized IRB protocol (WCG IRB protocol no. 20151664) and provided written informed consent to take part in the study. Participants of the UK Biobank study provided informed consent and ethical approval was provided by the UK’s National Health Service, National Research Ethics Service (Ethics Committee reference no. 11/NW/0382). Data analyses were conducted in accordance with the following research projects that have been deemed exempt under 45 CFR 46.104.d(4)(ii) by Institut Pasteur IRB: IRB-DB_2019-01 (SSC cohort), IRB2020-K-Exempt (UK Biobank) and IRB-DB_2019-03 (SFARI). The authors confirm that the manuscript complies with current policies on vulnerable groups and uses current language related to autism^58^.

### A note on terminology

Throughout the manuscript, we use the term ‘individuals with autism’ to refer to individuals who have a diagnosis of autism. This person first terminology is preferred by many but not all individuals with autism. We use the term ‘undiagnosed individuals’ to refer to parents and siblings of individuals with autism who do not have a diagnosis and individuals from the UK Biobank who also have not indicated that they have an autism diagnosis. We note that some of these individuals may have an autism diagnosis that is not recorded in the datasets used. We further note that some of these individuals may be autistic but may not have received a formal diagnosis.

### Samples

For the SSC, SPARKv1 and SPARKv2 cohorts, we downloaded genetic and clinical data from SFARI Base (https://sfari.org/sfari-base). For the SSC cohort, we selected 10,141 individuals with both WES and single-nucleotide polymorphism (SNP) array data, who were not twins and did not show a high number of erroneous variant calls (families filtered out, 12958, 14572 and 11037). For the SPARKv1 cohort, we selected 19,671 individuals with both WES and SNP array data, who were not withdrawn, not twins and not showing excessive number of variants or abnormal age, and from families in which both parents were undiagnosed and had available genetic data. For the SPARKv2 cohort, we selected 5,970 individuals with both WES and SNP array data, who were not withdrawn and from families in which both parents were undiagnosed and had available genetic data. For simplicity, the SPARKv1 and SPARKv2 samples were merged into one SPARK sample.

For the UK Biobank cohort, we downloaded genetic, demographic and brain imaging data from the UK Biobank database (project 18584). We selected 200,428 individuals with both WES and SNP array data, not twins (kinship < 0.4 from relationship file of UK Biobank) and who did not report autism-related symptoms (based on ICD10-F84 index or the autism diagnostic questionnaire).

For the aggregated iPSYCH sample, we downloaded tabular files for each gene of interest from the Autism Sequencing Consortium website (https://asc.broadinstitute.org/) and calculated the maximum allele numbers per status for all variants, corresponding to 4,811 individuals with autism and 5,214 undiagnosed individuals.

### Autism and constrained gene sets

We focused on coding exons of 220 autism-associated genes: genes from the SFARI Gene database with a score of 1 (https://gene.sfari.org/database/human-gene/), 102 genes from a recent case–control study of rare variations^8^ and 157 genes robustly associated with autism in multiple independent studies and unrelated individuals by the SPARK committee (http://sparkforautism.org) (Supplementary Table 1).

Constrained genes were defined based on suggested thresholds of the LoF observed/expected upper bound fraction < 0.35 or the probability of LoF intolerance > 0.9, both extracted from the gnomAD website (https://gnomad.broadinstitute.org)^13^.

The present study focused on autosomal genes and we filtered out the genes with an evidence of recessive type of inheritance^12^.

For sex-specific analyses of autism OR, all autism-associated genes on the X chromosome were also considered for male-specific analyses and only if they had no evidence of a recessive type of inheritance for female-specific analyses (dominant, *ARHGEF9*, *CASK*, *CDKL5*, *DDX3X*, *FMR1*, *HNRNPH2*, *IQSEC2*, *MECP2*, *NEXMIF*, *PCDH19* and *USP9X*; and recessive, *AFF2*, *ARX*, *ATRX*, *KDM5C*, *NLGN3*, *NLGN4X*, *PTCHD1*, *SLC9A6*, *SYN1* and *UPF3B*).

### Other neurodevelopmental and functional gene sets

Cognitive impairment, epilepsy and neurodevelopmental disorder genes were extracted from our previous work^12^. Briefly, cognitive impairment genes were those identified as ‘primary’ in the SysID database (https://sysid.cmbi.umcn.nl/), epilepsy genes extracted from six databases (The Lafora Gene Mutation Database, The Epilepsy Genetic Association Database, CarpeDB, EpilepsyGene, GenEpi and MeGene) and NDD genes from the Gene2Phenotype genes classified as associated with NDDs, restricted to those annotated as ‘brain’ or ‘cognition’.

Functional annotation of synaptic proteins were taken from SynGO^59^ and transcription proteins were extracted from Gene Ontology term transcription, DNA templated^60^.

Microcephaly and macrocephaly genes were extracted from our previous work^61^ and are available at https://genetrek.pasteur.fr/.

Gene coexpression modules in autism versus control brains were extracted from previous work by Voineagu et al.^24^. Module annotations to cell types were also extracted from this study.

### SNP arrays

For the SSC sample, the GRCh36-based SNP array data for the three different technologies (Illumina Omni1Mv1, *n* = 1,354; Omni1Mv3, *n* = 4,626; and Omni2.5, *n* = 4,240) were downloaded from SFARI Base (https://sfari.org/sfari-base) and 15 individuals were removed because they were twins. Arrays from each technology were mapped onto the GRCh37 human genome version separately. We downloaded the preprocessed GRCh37-based genotyping files of 26,879 SPARKv1 and 15,904 SPARKv2 participants from SFARI Base. SSC and SPARK genotyping files were filtered from ambiguous SNPs (A/T and G/C SNPs if minor allele frequency (MAF) > 0.4; SNPs with differing alleles; SNPs with >0.2 allele frequency difference; and SNPs not in reference panel) and imputed on the Haplotype Reference Consortium panel v.r1.1 (ref. ^62^) on the Michigan servers with default parameters^63^. GRCh37-based imputed genotyping files for 200,080 UK Biobank individuals were downloaded from the UK Biobank database (projects 51869 and 18584). After imputation we kept only variants with a *r*^2^ ≥ 0.8 and merged the three different SNP array technologies from the SSC sample keeping only SNPs shared between all three technologies.

### Admixture

We used the 1000 Genomes sequencing data of 2,504 individuals as a reference group of individuals of known ancestry^64^. We selected the 1000 Genomes SNPs that were present in the SSC, SPARKv1 and SPARKv2 datasets to perform a combined admixture for SFARI Base samples and 1000 Genomes SNPs that were present in the UK Biobank dataset to perform a separate admixture, using the Admixture v.1.3.0 tool^65^ on one to eight clusters. SSC, SPARKv1 and SPARKv2 genotypes, as well as UK Biobank genotypes, were projected on the corresponding admixture models based on 1000 Genomes data and we selected five clusters for separating the individuals by ancestry, corresponding to a low cross-validation error in both admixture models (Supplementary Fig. 1). Based on the reference EUR super-population, we used a fraction of each individual’s SNPs predicted as European ancestry threshold of ≥60% to define individuals as being of European ancestry, resulting in 8,067, 15,360, 4,346 and 188,856 individuals in SSC, SPARKv1, SPARKv2 and UK Biobank samples, respectively.

### Whole-exome sequences

We downloaded the GRCh37-aligned BAM files of 8,960 SSC participants from SFARI Base (https://sfari.org/sfari-base). We then called the variants using GATK v.3.8 following the Broad Institute Best Practices^66^ and lifted over all variants to the GRCh38 human genome version. We downloaded the preprocessed GRCh38-based pVCF files of 27,270 SPARKv1 and 16,004 SPARKv2 participants from SFARI Base. All functional-equivalent GRCh38-based pVCF files for 200,642 UK Biobank participants were downloaded from the UK Biobank database (projects 51869 and 18584). All variants from SSC, SPARK and UK Biobank samples were filtered for call rate > 0.9, genotype quality ≥ 30, depth > 20, allelic fraction ≥ 0.25 (and ≤0.75 for autosomal variants). Tabular lists of variants from the aggregated iPSYCH samples were downloaded from the Autism Sequencing Consortium website (https://asc.broadinstitute.org) and mapped to the GRCh38 human genome version (using chain file hg19toHg38.over.chain.gz).

We used VEP^67^ (using Ensembl 101) to annotate the variants. Non-neuro (individuals who were not cases of a few particular neurological disorders), non-Finnish European population frequencies were extracted using gnomAD exomes r2.1.1 (ref. ^13^). Variants with a MAF > 1%, present in >1% of each sample or affecting genes that were recurrently found mutated across different individuals in different families (*MUC4*, *MUC12*, *HLA-A*, *HLA-B*, *HYDIN*, *TTN*, *PAX5*, *OR2T10* and *MYH4*), were filtered out. We used Loftee^13^ to filter low-confidence variants or variants corresponding to ancestral alleles, as well as variants annotated with any flag by Loftee. All LoF variants affecting autism-associated genes were visually validated with Integrative Genomics Viewer^68^ on BAM/CRAM files for SSC, SPARK and UK Biobank samples.

We also performed further quality control for S-LoF annotation by visualizing the phase of variants for individuals carrying multiple nucleotide variants (MNVs) in the close vicinity of the originally reported S-LoF variants. Such MNVs, if in phase with the original S-LoF, could modify the effect of the variant on the encoded protein (changing from LoF to missense or synonymous variants). We filtered out 111 and 3,787 S-LoFs in autism-associated and constrained genes, representing 1.9% and 3.6% of the initial dataset, respectively.

For the independent regression analyses on autism status in the iPSYCH sample, we performed additional quality control (QC) steps on the 236 S-LoFs in autism-associated genes and 1,345 S-LoFs in constrained genes. The initial QC steps for the iPSYCH Danish Blood Spot WES data have been described previously^69^. Briefly, after the first round of sample-level and variant-level QC, three call-rate filters were used subsequently, (1) remove variants with a call rate < 90%; (2) remove samples with a call rate < 95%; and (3) remove variants with a call rate < 95%. Between the sample call-rate filter and the final variant call-rate filter, one of each pair of related samples (relatedness as a pi-hat value ≥ 0.2) was removed. Subsequently, we selected for this study the individuals diagnosed with autism no later than by the end of 2016. This gave us a study sample of 4,622 cases and 4,753 undiagnosed individuals. We defined rare variants as having an allele count no greater than five across our dataset (*n* = 9,375) and the non-Finnish Europeans from non-psychiatric exome subset of the gnomAD (*n* = 44,779). We matched these S-LoFs to the original S-LoFs and identified 138 out of 236 S-LoFs in autism-associated genes and 767 out of 1,345 S-LoFs in constrained genes in iPSYCH. Replication analyses were based on these S-LoFs.

### Relative position on encoded protein and pext score

We annotated the relative position of the variants on the encoded protein using the Loftee coding sequence (CDS) position when available or VEP CDS position otherwise and the CDS size for each transcript from BioMart (https://www.ensembl.org/biomart/martview/). To measure exon usage in different isoforms of each gene within brain tissues, we downloaded the base-level pext score from the gnomAD website (https://gnomad.broadinstitute.org)^15^. Briefly, the pext score summarizes the isoform expression values across tissues and allows measurement of the expression status of exonic regions across tissues, at the exon level. For each exon of each gene, we selected the maximum value of the pext measures from 13 brain tissues (amygdala, anterior cingulate cortex BA24, caudate basal ganglia, cerebellar hemisphere, cerebellum, cortex, frontal cortex BA9, hippocampus, hypothalamus, nucleus accumbens basal ganglia, putamen basal ganglia, spinal cord and substantia nigra). For splice-site variants, we measured the relative position and pext score based on the closest coding exon (position of the variant ±3 bp). We finally filtered variants using the pext score, reflecting how much the corresponding exon was expressed in brain tissues.

### Gene-level autism odds ratio

The autism OR was measured to estimate the strength of the association between outcome (autism diagnostic) and genetic risk factors (carrying an LoF variant) for each gene, using the following formula:

$${\rm{autism}}\; {\rm{OR}}=\frac{{n{{\_}}\rm{carriers}}_{\rm{diagnosed}}\times {n{{\_}}\rm{noncarriers}}_{\rm{undiagnosed}}}{{n{{\_}}\rm{carriers}}_{\rm{undiagnosed}}\times {n{{\_}}\rm{noncarriers}}_{\rm{diagnosed}}}$$

Given the large difference in sample size between diagnosed and undiagnosed individuals and given that the definition of rarity of variants depends on the sample size, we performed 100 iterations of a sub-sampling procedure: (1) randomly selecting as many undiagnosed individuals as diagnosed individuals and (2) selecting singletons among diagnosed individuals and among undiagnosed individuals separately. We then used the average number of carriers among undiagnosed individuals to estimate the autism OR for each gene. To compare the autism OR to what would be expected by chance given our samples, we also performed a bootstrapping procedure, randomly selecting as many individuals as diagnosed individuals, artificially labeling them as diagnosed and labeling the rest of the sample as undiagnosed and measuring the autism OR using the same algorithm. We ran this procedure 10,000 times, measured for each gene the number of times (*M*) the expected autism OR was higher or equal to the observed autism OR, divided it by the number of bootstraps performed (*N*) and used the (*M* + 1) / (N + 1) ratio as an empirical *P* value. The 95% CI around this empirical *P* value was measured using the following formula to assess the degree of certainty of the empirical *P* value:

$$95 \%\, {\rm{CI}}=P\pm 1.96\times \sqrt{\frac{P\times (1-P)}{N+1}}$$

We verified that all reported signals for the analyses described in the manuscript were similar when restricting the analyses to genes with autism ORs significantly higher than expected by chance (upper fraction of the 95% CI of the empirical *P* value < 0.05), with the exception of the significance of the brain anatomy results that were insufficiently powered.

### Developmental brain gene expression

The developmental brain transcriptome data from 42 specimen and up to 16 brain structures were downloaded from the Allen Brain Atlas BrainSpan database (https://www.brainspan.org/). Only expression reads per kilobase of exon model per million mapped reads values >1 were considered for expression analysis. Values for each gene were averaged across four brain regions and eight developmental periods as previously described^23^. Brain regions were defined as follows: R1, posterior inferior parietal cortex, primary auditory cortex, primary visual cortex, superior temporal cortex, inferior temporal cortex; R2, primary somatosensory cortex, primary motor cortex, orbital prefrontal cortex, dorsolateral prefrontal cortex, medial prefrontal cortex, ventrolateral prefrontal cortex; R3, striatum, hippocampus, amygdala; and R4, mediodorsal nucleus of the thalamus, cerebella cortex. Developmental periods were defined as follows: P1, early fetal; P2, early mid-fetal; P3, late mid-fetal; P4, late fetal; P5, infancy; P6, childhood; P7, adolescence; and P8, young adult. Note that only one individual was available for P1R4 in the BrainSpan database; the corresponding period/region was therefore not investigated in this study. For the analysis of the correlation between gene expression and autism OR, we artificially replaced infinite autism OR values by the highest measurable autism OR in the gene set and the Pearson correlation test was performed in the log_10_ space for both expression and OR of autism-associated genes.

### Autism polygenic score computation

SSC, SPARKv1, SPARKv2 and UK Biobank imputed genotyping data were filtered separately from variants absent from >1% of individuals (geno001 parameter), then variants present in all four samples were merged with PLINK v.1.9 (ref. ^70^). The PGS for autism was computed by using the GWAS summary statistics from iPSYCH and the Psychiatric Genomics Consortium (PGC)^2^. To exclude overlap in participants from the test and discovery data in the PGS analysis, the GWAS meta-analysis summary statistics reported^2^ were recalculated with the SSC data excluded. We used the SBayesR^71^ method of the GCTB tool v.2.02 with the banded linkage disequilibrium matrix and suggested options (https://cnsgenomics.com/software/gctb) on the PGC-ASD summary statistics to estimate the posterior statistics of SNP effects. We finally computed the autism PGS using PLINK v.1.9 based on SBayesR-derived statistics for common SNPs (MAF > 10%).

We performed a principal-component analysis using PLINK v.2.0 and extracted the four first principal components to control for population structure when using the autism PGS in regression analyses.

We also calculated autism PGS values for subsets of genes. First, we selected the SNPs that fall in a window of ±20 kb from the minimum protein-coding transcript start and stop, to calculate the gene-specific autism PGS. Transcript start and stop positions were based on Ensembl annotation v.107. Next, we further selected subsets of the protein-coding genes corresponding to those present in the lists of autism-associated genes, constrained genes, SynGO genes or micro- or macrocephaly genes. All numbers are reported in Supplementary Fig. 4.

For the iPSYCH replication sample, we used our best genetic predictor as measure of common variant load, which is generated in part internally through a 50-fold cross-validation process, where the full iPSYCH2015 sample^72^ was pruned for related individuals (at pi-hat 0.2) and split at random in 50 subsets of almost equal size. For each subset, the index subset, a GWAS was run on the complement using PLINK v.1.9. The results were then meta-analyzed using METAL^73^ with the PGC summary statistics for autism^2^. The resulting summary statistics were filtered for MAF 1% and info-score 0.9 and transformed using LDpred2 to create a PGS on the index subset^74^.

### Psychiatric, developmental, cognitive and socioeconomic data

The SCQ results for SSC and SPARK samples were downloaded from SFARI Base (https://sfari.org/sfari-base) and were available for 8,235 probands and 4,176 non-autistic siblings of European ancestry. Sex assigned at birth was available for 19,706 individuals from the SPARK sample and 7,809 individuals from the SSC sample. The autism factors and IQ score bins for SSC and SPARK samples were available for 4,180 probands from a previous study^7^. Briefly, in the SPARK study, full-scale IQ scores were available based on parent reports on ten IQ score bins: <25, 25–39, 40–54, 55–69, 70-79, 80–89, 90–109, 110–119, 120–129 and >130. For the SSC samples, full-scale IQ scores were converted into IQ bins to match what was available from the SPARK study^7^. The resulting IQ score bins were treated as continuous variables. The developmental milestones for SPARK samples were downloaded from SFARI Base (https://sfari.org/sfari-base) and were available for 4,722 probands. The number of developmental disorders was available for 6,910 SPARK individuals, including 5,630 individuals with autism.

For the independent iPSYCH replication cohort, sex was extracted from the Danish registry database, corresponding to biological sex. The diagnoses of autism and cognitive impairment were conferred by the end of 2016 based on the psychiatric central register. We used the ICD10 codes F70–F79 for cognitive impairment diagnoses. There were 1,017 individuals diagnosed with both autism and cognitive impairment (with IQ < 70) and 3,605 individuals with autism only (with IQ ≥ 70).

For the UK Biobank individuals, age when attending assessment center and genetic sex were available for all 188,856 unselected European individuals. The fluid intelligence test is a simple unweighted sum of the number of correct answers given to the 13 fluid intelligence questions and was completed by 112,614 individuals. More information on the touch-screen fluid intelligence test, along with the questions asked, is available at the UK Biobank website (https://biobank.ndph.ox.ac.uk/showcase/refer.cgi?id=100231). A comparative analysis of this test and other reference tests has been performed^75^. We used the highest qualification an individual had achieved (for example university/college degree and A levels), excluded participants with only ‘other professional qualifications’ and those who did not provide an answer to this question, retaining data for 156,483 individuals and categorizing in five bands (Certificate of Secondary Education (CSEs) or equivalent, O levels/General Certificate of Secondary Education (GCSEs) or equivalent, National Vocational Qualification (NVQ) or Higher National Diploma (HND) or Higher National Certificate (HNC) or equivalent, A levels/AS levels or equivalent and college or university degree). Annual income was categorized by the UK Biobank sample in five bands (<£18,000, £18,000–30,999, £31,000–51,999, £52,000–100,000 and >£100,000) and was available for 162,968 participants. The Townsend deprivation index is a measure of material deprivation within a population, assigned to each individual as a score corresponding to the output area in which their postcode is located and was available for 188,630 individuals.

For brain anatomy analyses, early-life trauma variables were downloaded from the UK Biobank database. Whether individuals were adopted with a yes/no answer was available for 188,443 individuals and whether individuals felt loved, felt hated, were physically abused by family or had someone to take them to doctor when needed as a child for 65,104 individuals. We excluded participants who responded ‘do not know’ or ‘prefer not to answer’ to these questions.

For participation analyses of qualification level, we considered as respondent participants who answered ‘other professional qualifications’, ‘CSEs or equivalent’, ‘O levels/GCSEs or equivalent’, ‘NVQ or HND or HNC or equivalent’, ‘A levels/AS levels or equivalent’ or ‘college or university degree’. For participation analyses of income, we considered as respondent participants who answered ‘<£18,000’, ‘£18,000–30,999’, ‘£31,000–51,999’, ‘£52,000–100,000’ and ‘>£100,000’.

### Phenome-wide association study in UK Biobank

We performed a phenome-wide association study of 18,224 phenotypes present in the UK Biobank database (listed in Supplementary Table 6), for a total of 188,736 individuals. We used the PHESANT software (https://github.com/MRCIEU/PHESANT)^76^ with default parameters and presence of a S-LoF in an autism-associated gene as a trait of interest (binary trait with ‘genetic = TRUE’ and ‘standardize = FALSE’ arguments). Each regression analysis used sex (National Health Service recorded or self-reported), age at recruitment and type of array (BiLEVE or Axiom) as covariates. We extracted the *β* coefficients from the combined result output, as well as *P* values that were further corrected for multiple testing using the FDR method. *β* coefficients for the following traits were reversed so that lower levels were indicated with a negative sign: ‘qualifications’, ‘alcohol intake frequency’, ‘education score (England)’, ‘employment score (England)’, ‘health score (England)’ and ‘income score (England)’.

### Brain structural anatomy

Imaging-derived phenotype (IDP) data were downloaded from the UK Biobank database (projects 40980 and 18584). A total of 68 metrics for cortical regions and 16 metrics for subcortical regions, calculated using FreeSurfer and FSL software using the Desikan–Killiany Atlas, were provided for 21,040 individuals with genetic data. Details of the acquisition protocol and imaging processing toolbox are available on the UK Biobank website at https://biobank.ctsu.ox.ac.uk/crystal/crystal/docs/brain_mri.pdf. Four global IDPs were investigated: total cortical volume, total cortical thickness, total cortical surface area and total subcortical volume. The total brain IDPs were obtained by summing left and right hemisphere global measures. Carriers of S-LoFs have a slightly lower age distribution compared to non-carriers in the subsample with imaging data available, although both are in the 40–70-year age range (*P* = 0.015, Mann–Whitney *U*-test).

### Multivariable regression analyses

We performed ordinal logistic regression analyses for autism status using the below formula. The same formula was used for autism status and cognitive impairment in the iPSYCH replication sample.

$$\begin{array}{rcl}{\mathrm{Response}} & \sim & {{{\beta}}}_{0}+{{{\beta}}}_{1}{\mathrm{LoF}}+{{{\beta}}}_{2}{\mathrm{PGS}}+{{{\beta}}}_{3}{\mathrm{sex}}+{{{\beta}}}_{4}{\mathrm{LoF}}\times {\mathrm{PGS}}\\&& +\,{{{\beta}}}_{5}{\mathrm{PC}}1+{{{\beta}}}_{6}{\mathrm{PC}}2+{{{\beta}}}_{7}{\mathrm{PC}}3+{{{\beta}}}_{8}{\mathrm{PC}}4+\varepsilon\end{array}$$

We performed linear regression analyses for SCQ *t*-score, IQ score bins, autism factors and developmental milestones on individuals with autism using the following formula:

$$\begin{array}{rcl}{\mathrm{Response}} & \sim & {{{\beta}}}_{0}+{{{\beta}}}_{1}{\mathrm{LoF}}+{{{\beta}}}_{2}{\mathrm{PGS}}+{{{\beta}}}_{3}{\mathrm{sex}}+{{{\beta}}}_{4}{\mathrm{LoF}}\times {\mathrm{PGS}}\\&&+\,{{{\beta}}}_{5}{\mathrm{PC}}1+{{{\beta}}}_{6}{\mathrm{PC}}2+{{{\beta}}}_{7}{\mathrm{PC}}3+{{{\beta}}}_{8}{\mathrm{PC}}4+\varepsilon\end{array}$$

We performed linear regression analyses for fluid intelligence score and Townsend deprivation index on UK Biobank individuals using the following formula:

$$\begin{array}{rcl}{\mathrm{Response}} & \sim & {{{\beta}}}_{0}+{{{\beta}}}_{1}{\mathrm{LoF}}+{{{\beta}}}_{2}{\mathrm{PGS}}+{{{\beta}}}_{3}{\mathrm{sex}}+{{{\beta}}}_{4}{\mathrm{LoF}}\times {\mathrm{PGS}}\\&& +\,{{{\beta}}}_{6}{\mathrm{age}}+{{{\beta}}}_{7}{\mathrm{PC}}1+{{{\beta}}}_{8}{\mathrm{PC}}2+{{{\beta}}}_{9}{\mathrm{PC}}3+{{{\beta}}}_{10}{\mathrm{PC}}4+\varepsilon\end{array}$$

We performed ordinal logistic regression analyses for income and qualification level on UK Biobank individuals.

$$\begin{array}{rcl}{\mathrm{Response}} & \sim & {{{\beta}}}_{1}{\mathrm{LoF}}+{{{\beta}}}_{2}{\mathrm{PGS}}+{{{\beta}}}_{3}{\mathrm{sex}}+{{{\beta}}}_{4}{\mathrm{LoF}}\times {\mathrm{PGS}}\\&&+\,{{{\beta}}}_{6}{\mathrm{age}}+{{{\beta}}}_{7}{\mathrm{PC}}1+{{{\beta}}}_{8}{\mathrm{PC}}2+{{{\beta}}}_{9}{\mathrm{PC}}3+{{{\beta}}}_{10}{\mathrm{PC}}4+\varepsilon\end{array}$$

For brain anatomy among UK Biobank individuals, multivariable linear regressions were performed separately for global cortical thickness, surface area, volume and subcortical volume *z*-scored IDPs with the following formula, with the site variable representing the location where the scan was performed:

$$\begin{array}{rcl}{\mathrm{Response}} &\sim& {{{\beta}}}_{0}+{{{\beta}}}_{1}{\mathrm{LoF}}+{{{\beta}}}_{2}{\mathrm{PGS}}+{{{\beta}}}_{3}{\mathrm{sex}}+{{{\beta}}}_{4}{\mathrm{age}}+{{{\beta}}}_{5}{\mathrm{LoF}}:{\mathrm{PGS}}\\&&+\,{{{\beta}}}_{6}{\mathrm{LoF}}:{\mathrm{sex}}+{{{\beta}}}_{7}{\mathrm{LoF}}:{\mathrm{age}}+{{{\beta}}}_{8}{\mathrm{sex}}:{\mathrm{age}}+{{{\beta}}}_{9}{{\mathrm{age}}}^{2}+{{{\beta}}}_{10}{\mathrm{site}}+\varepsilon\end{array}$$

Multivariable linear regressions were performed separately for each 68 cortical regions and 16 subcortical regions using the following formula, adding the total measure for each metric (for example global cortical volume for the volume of the 68 cortical regions) as a covariate:

$$\begin{array}{rcl}{\mathrm{Response}}& \sim & {{{\beta}}}_{0}+{{{\beta}}}_{1}{\mathrm{LoF}}+{{{\beta}}}_{2}{\mathrm{PGS}}+{{{\beta}}}_{3}{\mathrm{sex}}+{{{\beta}}}_{4}{\mathrm{age}}\\&&+\,{{{\beta}}}_{5}{\mathrm{LoF}}:{\mathrm{PGS}}+\,{{{\beta}}}_{6}{\mathrm{LoF}}:{\mathrm{sex}}+{{{\beta}}}_{7}{\mathrm{LoF}}:{\mathrm{age}}\\&&+\,{{{\beta}}}_{8}{\mathrm{sex}}:{\mathrm{age}}+{{{\beta}}}_{9}{{\mathrm{age}}}^{2}+{{{\beta}}}_{10}{\mathrm{site}}+{{{\beta}}}_{11}{\mathrm{total}}+\varepsilon\end{array}$$

Multivariable regressions on brain anatomy were also performed with early-life trauma and Townsend deprivation index as covariates, using the following formula:

$$\begin{array}{rcl}{\rm{Response}} & \sim & {\beta}_{0}+{\beta}_{1}{\rm{LoF}}+{\beta}_{2}{\rm{PGS}}+{\beta}_{3}{\rm{sex}}+{\beta}_{4}{\rm{age}}+{\beta}_{5}{\rm{LoF}}:{\rm{PGS}}\\&& +\,{\beta}_{6}{\rm{LoF}}:{\rm{sex}}+{\beta}_{7}{\rm{LoF}}:{\rm{age}}+{\beta}_{8}{\rm{sex}}:{\rm{age}}+{\beta}_{9}{\rm{age}}^{2}\\ &&+\,{\beta}_{10}{\rm{site}} +\,{\beta}_{11}{\rm{trauma}}/{\rm{Townsend}}\,{\rm{index}}\\ &&+\,{\beta}_{12}{\rm{LoF}}:{\rm{trauma}}/{\rm{Townsend}}\,{\rm{index}}\\ && +\, {\beta}_{13}{\rm{PGS}}:{\rm{trauma}}/{\rm{Townsend}}\,{\rm{index}}\,(+{\beta}_{14}{\rm{total}})+\varepsilon \end{array}$$

For regressions not involving brain anatomy, PC1–4 represent the first four principal components of the principal-component analysis based on genotyping data. Results were presented as standardized *β* coefficients. To evaluate the significance of results, we used the Benjamini–Hochberg FDR method for *P* value correction. Multiple testing correction was applied separately for each covariate and independently for (1) autism status, SCQ *t*-score, IQ score bins and autism factors; (2) developmental milestones; and (3) socioeconomic and fluid intelligence features. For multivariable analyses of brain anatomy, multiple testing correction was applied to all regressions together.

For the estimation of the effect size of S-LoFs on socioeconomic status among UK Biobank individuals, we used the linear regressions described above for fluid intelligence score and Townsend deprivation index. For income, we assigned with each category the midpoint of the range: <£18,000 = £15,000; £18,000–30,999 = £24,500; £31,000–51,999 = £41,500; £52,000–100,000 = £76,000; and >£100,000 = £150,000. For education years, we assigned years of completion to each qualification level as follows: CSEs or equivalent = 0 years; O levels/GCSEs or equivalent = 2 years; NVQ or HND or HNC or equivalent = 2 years; A levels/AS levels or equivalent = 3 years; and college or university degree = 6 years. All linear regressions used to estimate the effect of S-LoFs used the following formula:

$$\begin{array}{rcl}{\rm{Response}} & \sim & {{{\beta}}}_{0}+{{{\beta}}}_{1}{\mathrm{LoF}}+{{{\beta}}}_{2}{\mathrm{PGS}}+{{{\beta}}}_{3}{\mathrm{sex}}+{{{\beta}}}_{4}{\mathrm{LoF}}\times {\mathrm{PGS}}+{{{\beta}}}_{6}{\mathrm{age}}\\&&+\,{{{\beta}}}_{7}{\mathrm{PC}}1+{{{\beta}}}_{8}{\mathrm{PC}}2+{{{\beta}}}_{9}{\mathrm{PC}}3+{{{\beta}}}_{10}{\mathrm{PC}}4+\varepsilon\end{array}$$

### Statistical analyses

Most of the statistical analyses in this work were performed using statistical test implementations from Python libraries scipy^77^ and statsmodels^78^. If not otherwise stated, analyses, including adjusting *P* values for multiple testing, used the Benjamini–Hochberg control for FDR^79^.

### Reporting summary

Further information on research design is available in the Nature Portfolio Reporting Summary linked to this article.

## Online content

Any methods, additional references, Nature Portfolio reporting summaries, source data, extended data, supplementary information, acknowledgements, peer review information; details of author contributions and competing interests; and statements of data and code availability are available at 10.1038/s41591-023-02408-2.

## Supplementary information

Supplementary Information Supplementary Figs. 1–6.

Reporting Summary

Supplementary Tables 1–9 Supplementary Tables 1–9.

## Data Availability

Researchers can obtain the whole-exome and SNP genotyping data from the SSC and SPARK cohorts used in this study by applying at https://base.sfari.org. The UK Biobank whole-exome, SNP genotyping, phenotypic and brain imaging data can be obtained by applying at the UK Biobank database (https://www.ukbiobank.ac.uk/). The human neurodevelopmental transcriptome dataset is available on the BrainSpan database (http://www.brainspan.org). Functional annotations can be obtained from SynGO (https://syngoportal.org/) and Gene Ontology (http://current.geneontology.org/annotations/goa_human.gaf.gz). Human reference genomes were obtained from https://www.ncbi.nlm.nih.gov/grc/human. Electronic health records and healthcare claims data used in the present study for the UK Biobank individuals are not publicly available due to patient privacy concerns. Prevalence and autism OR measures can be visualized and downloaded on https://genetrek.pasteur.fr/.

## Code availability

Code used to implement the post-processing analyses in this paper is available at https://github.com/thomas-rolland/subdiagnostic-autism-variants.
