# Supplementary material for: Phenotypic effects of genetic variants associated with autism
Source: Nat Med. 2023 Jun 26;29(7):1671–80. doi: 10.1038/s41591-023-02408-2 (PMC10353945; doi:10.1038/s41591-023-02408-2)
Supplement: Supplementary file 1 — Supplementary Figs. 1–6. [file 41591_2023_2408_MOESM1_ESM.pdf]

nature medicine

Article

https://doi.org/10.1038/s41591-023-02408-2

Phenotypic effects of genetic variants associated with autism

In the format provided by the authors and unedited

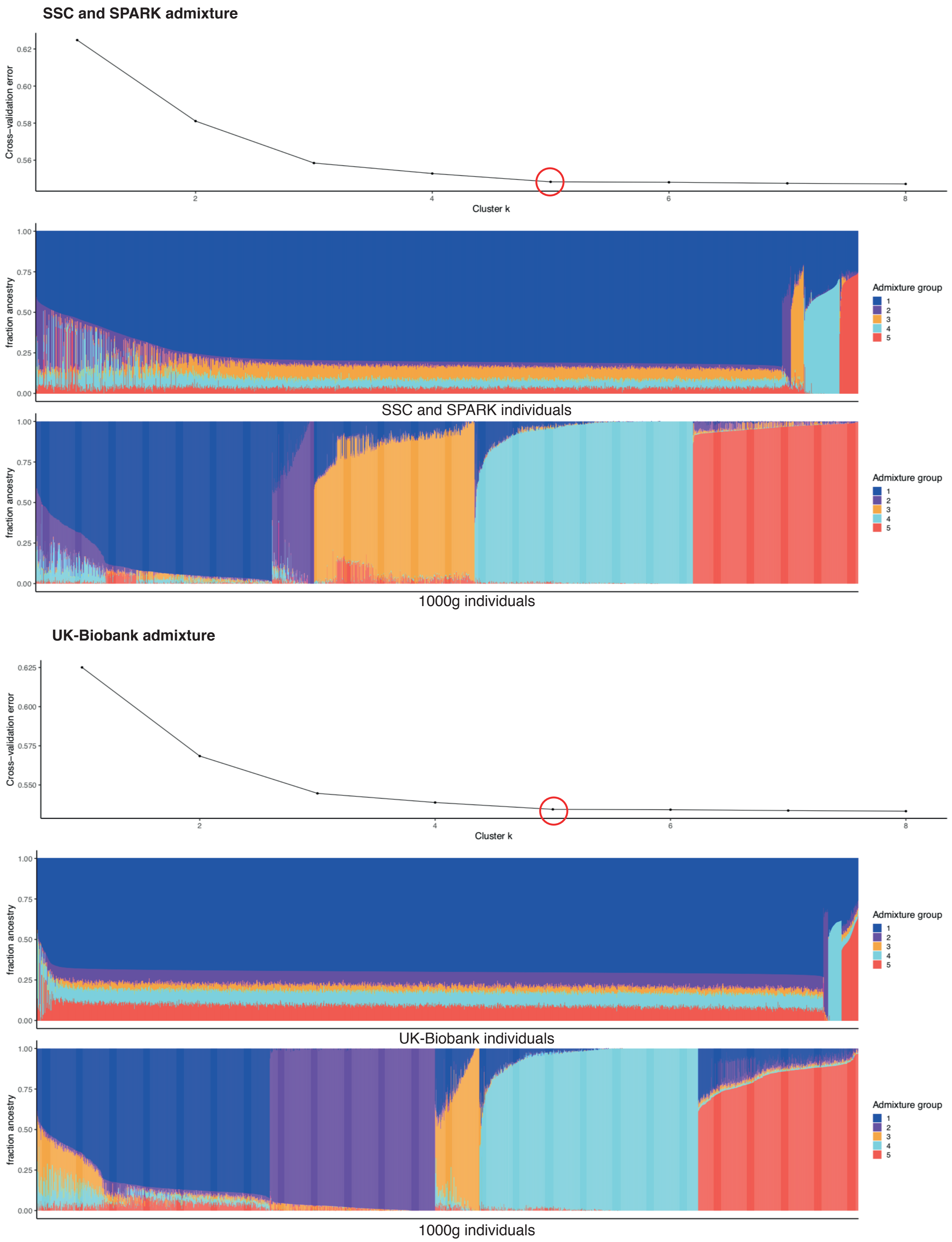

Supplementary Figure 1. Admixture results for UK-Biobank, SSC and SPARK individuals.
For each cohort, the cross-validation errors were shown for increasing values of clusters, and the resulting fraction of ancestry predicted in each admixture group was shown for five clusters. SSC and SPARK individuals were merged for this analysis. Fractions were shown for reference population individuals and for individuals of each cohort. The predicted European group, used for subsequent prediction of European ancestry (individuals with > 60% predicted fraction of European ancestry were considered European, see Methods), was shown in dark blue.

Meta-analysis n_{ASD} = 13,091, $n_{relatives+controls}$ = 213,558

Cohort	UK-Biobank	SSC	SPARK	iPSYCH
Probands	-	2,041	6,239	4,811
Siblings	-	1,944	2,344	-
Parents	-	4,082	11,118	-
Controls	188,856	-	-	5,214

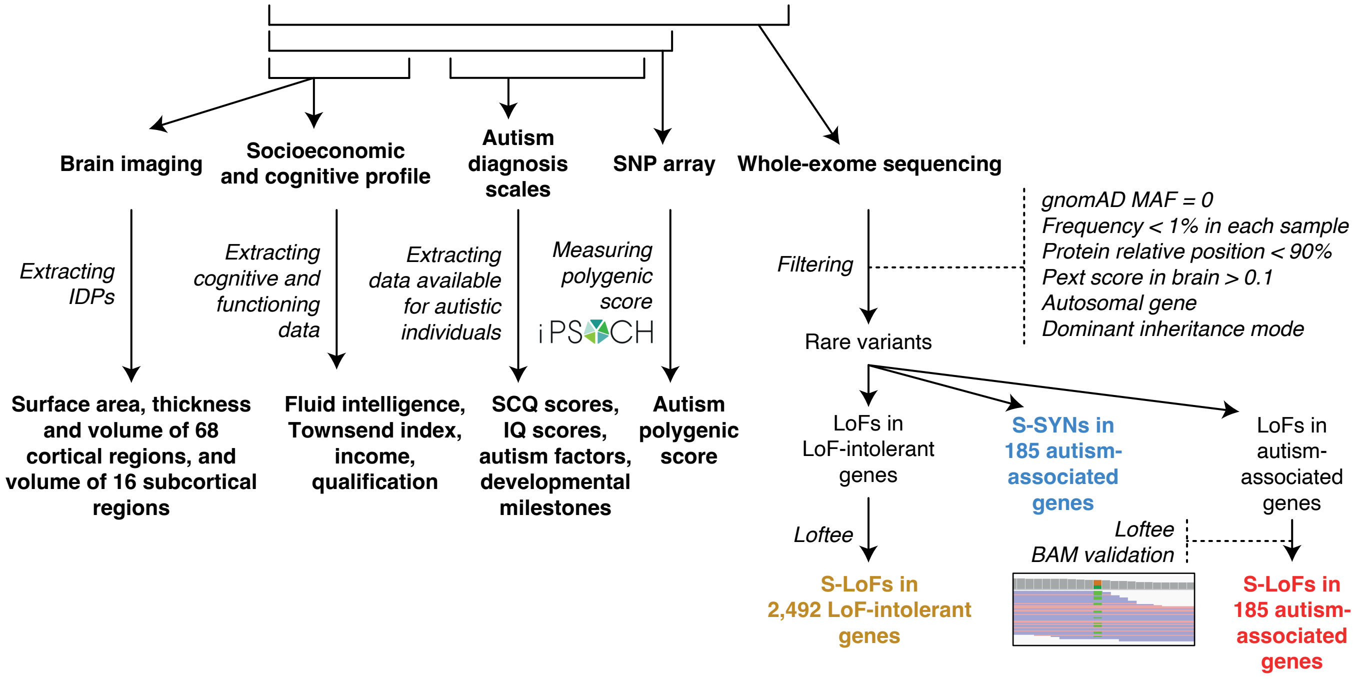

Supplementary Figure 2. Framework of the study.

The schematic represents the different analyses performed in the manuscript, on all or subsets of the 13,091 diagnosed and 213,558 undiagnosed individuals with both whole-exome sequencing data available. Brain imaging and cognitive and functioning data were available only for a subset of the UK-Biobank individuals. SCQ t-scores, IQ scores bins, autism factors and developmental milestones were extracted only for a subset of diagnosed individuals from the SSC and SPARK samples. SNP array data were available for all individuals except from the iPSYCH cohort, which was treated separately (Methods).

SHANK3

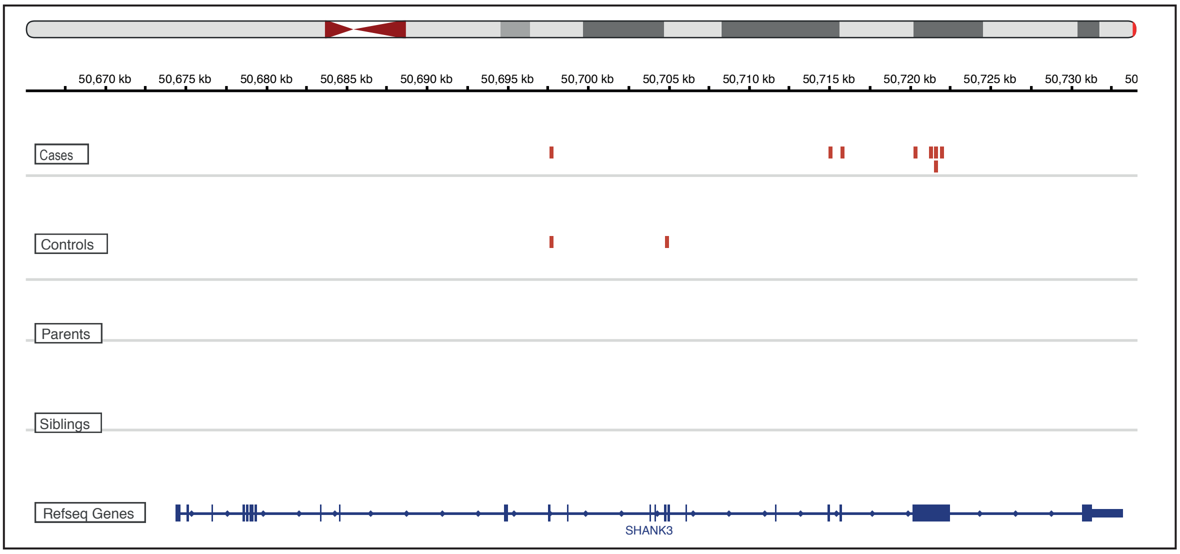

NRXN2

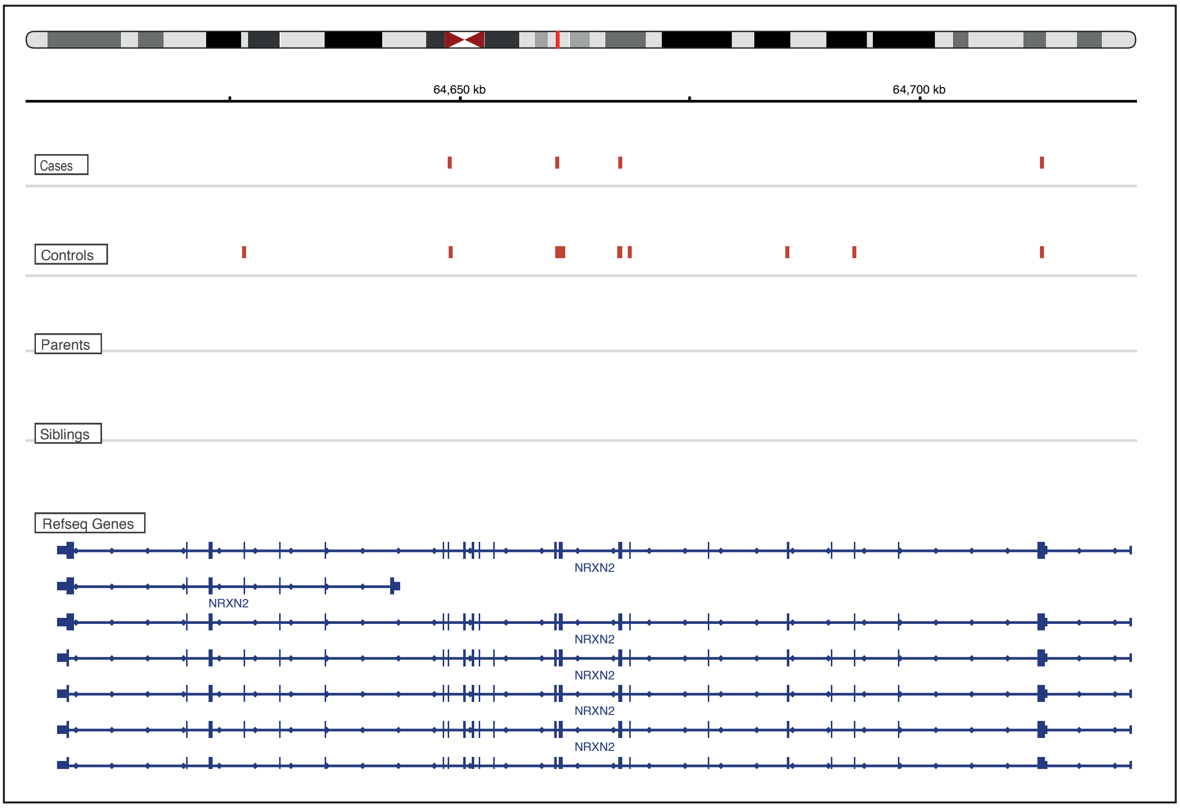

Supplementary Figure 3. Examples of variants mapping to exons of autism-associated genes. For SHANK3 and NRXN2, the variants identified in diagnosed and undiagnosed individuals are indicated on the Genetrek website. The four tracks correspond to variants identified in 13,091 autistic individuals, 194,070 undiagnosed individuals, 15,200 parents of autistic individuals, and 4,288 non-autistic siblings of autistic individuals. For each track, the first line represents the variants identified, colored by variant type (S-LoF in autism-associated gene in red, S-LoF in constrained gene in orange, S-SYN in autism-associated gene in blue). The four other lines inform about the sex and inheritance mode of the identified variant (order: de novo in female, not de novo - "other" - in female, de novo in male, other in male), with grey marks corresponding to homozygous reference allele and blue marks heterozygous allele.

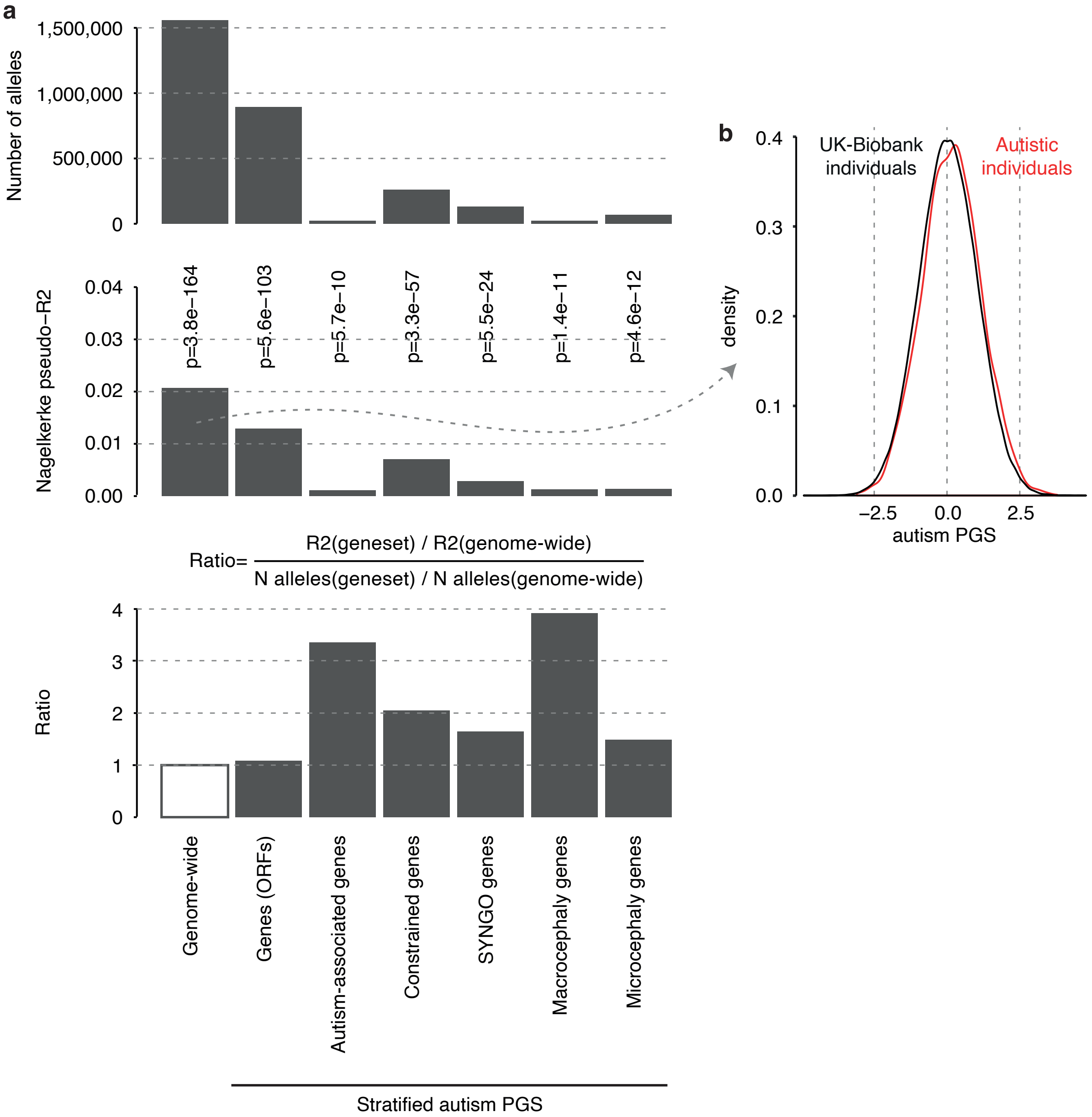

Supplementary Figure 4. Calculation of the autism PGS.

(**a**) The autism PGS was calculated based on all SNPs in the autism GWAS summary statistics (genome-wide autism PGS) and on the subset of SNPs in genic regions (see Methods). We further measured the autism PGS stratified by gene sets including autism-associated genes, constrained genes, synaptic genes from the SynGO database, macrocephaly and microcephaly genes. For each autism PGS calculation, the total number of alleles used, the Nagelkerke's pseudo-R2 correlation coefficient for the autism PGS comparing autistic individuals and UK-Biobank individuals and p-value from a Chi-squared likelihood ratio test comparing the model including the autism PGS to the null model including only the first four principal components of the PCA based on genotyping data, and the ratio between the pseudo-R2 and the number of alleles used are shown. (**b**) Density plots of genome-wide autism PGS scores for autistic and UK-Biobank individuals.

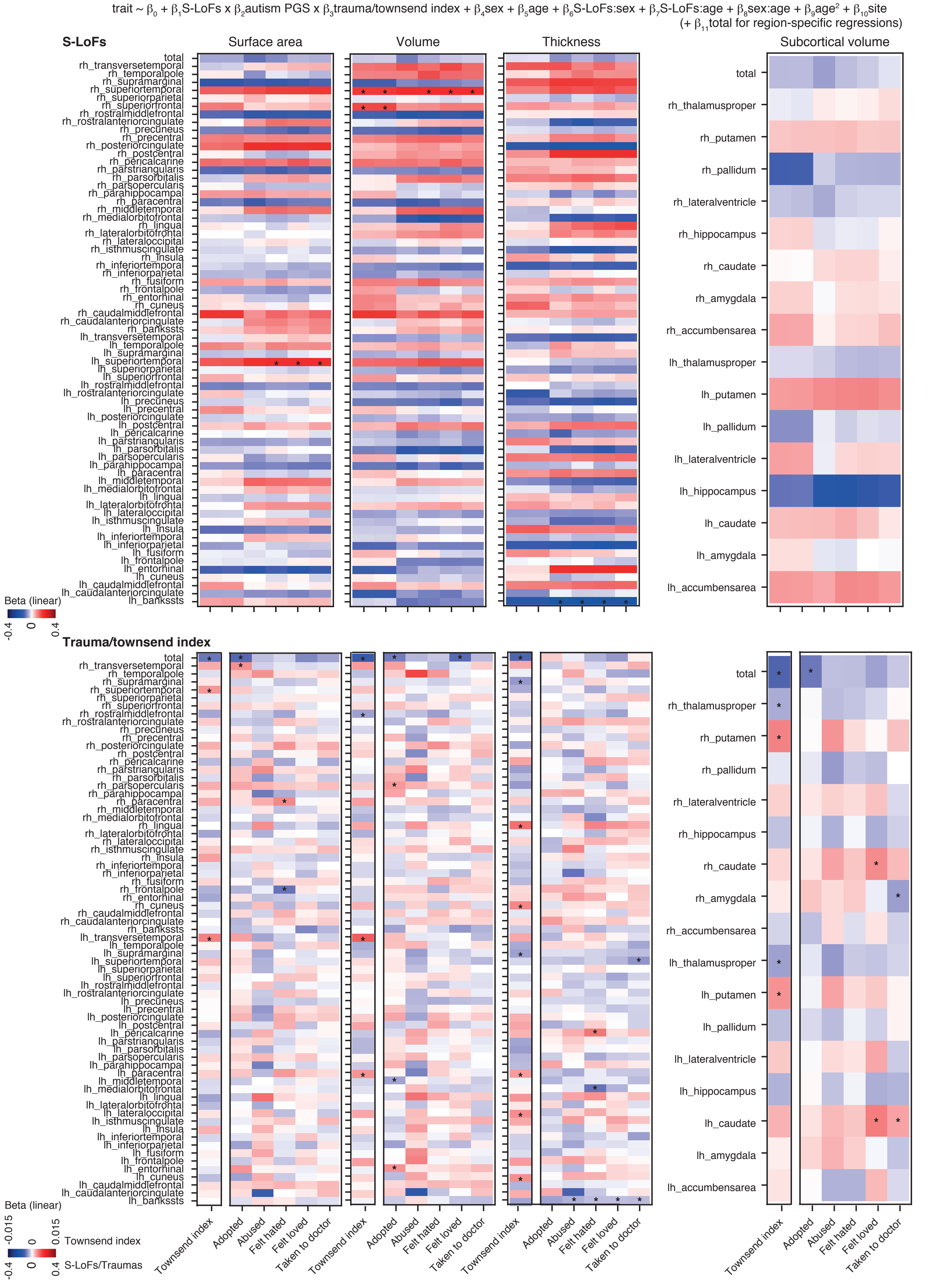

Supplementary Figure 5. Effect of early life trauma and Townsend index on regression results for brain anatomy.
Heatmaps representing the standardized beta values associated with S-LoFs (top) or early life trauma/Townsend index (bottom) for brain anatomy traits. Total values for cortical thickness, surface area and volume were measured as the sum of all 68 regions (Methods). S-LoFs in autism-associated and constrained genes were grouped to increase sample size. Each column represents a regression analysis for one trait, alternatively adding early life trauma or Townsend index covariates (see complete results in Supplementary Table 7). P-values were corrected for multiple testing using the FDR method (* correspond to corrected p-values < 0.05).

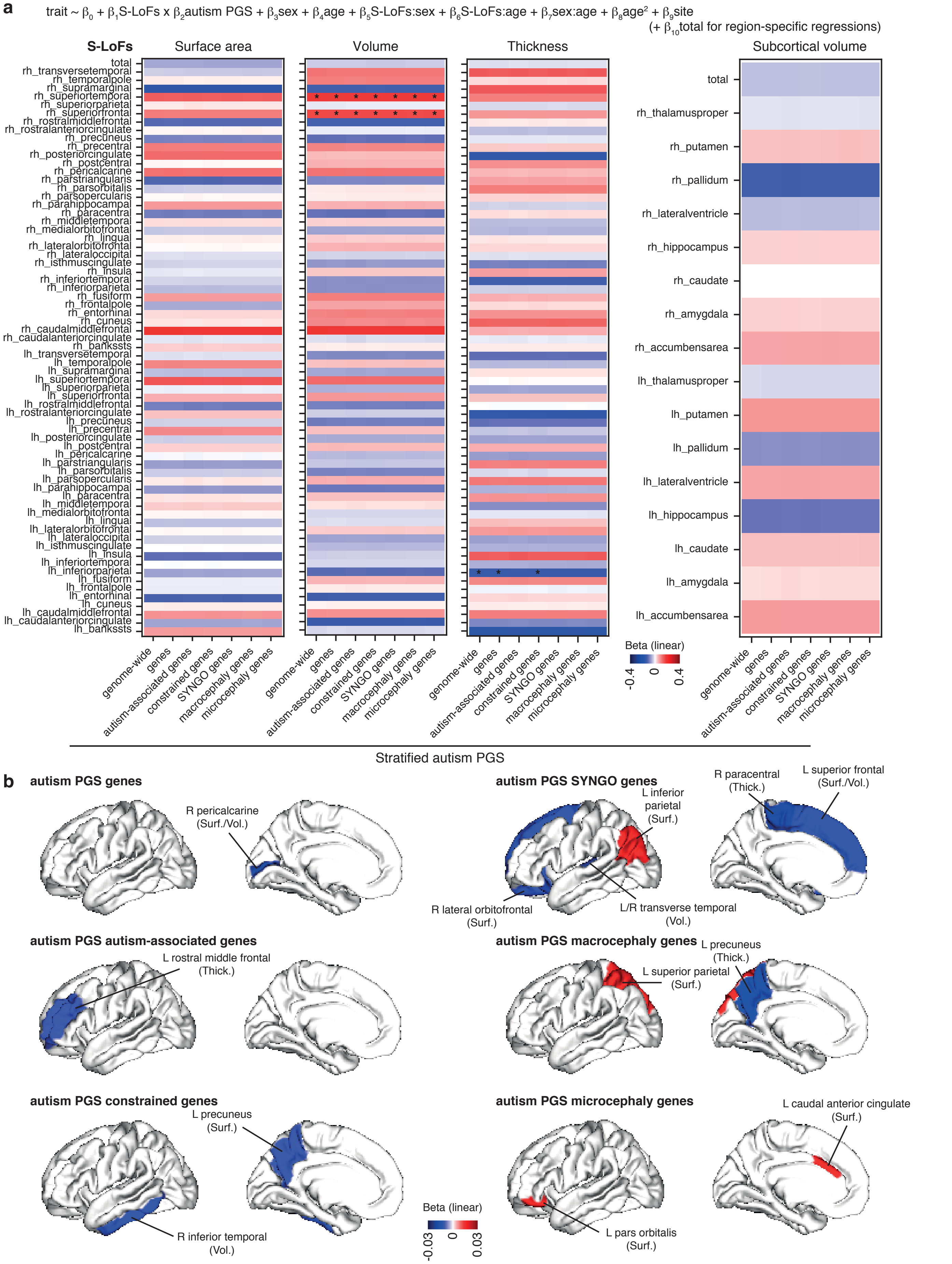

Supplementary Figure 6. Regression analysis of stratified autism PGS for brain anatomy.
(**a**) Heatmap representing the standardized beta values associated with S-LoFs for brain anatomy traits. Total values for cortical thickness, surface area and volume were measured as the sum of all 68 regions (Methods). S-LoFs in autism-associated and constrained genes were grouped to increase sample size. Each column represents a regression analysis for one trait, alternatively using different versions of the autism PGS as covariates (see complete results in Supplementary Table 7). P-values were corrected for multiple testing using the FDR method (* correspond to corrected p-values < 0.05). (**b**) Brain maps showing the beta coefficients associated to autism PGS from multivariable regression analyses of brain sub-regions using different versions of the autism PGS. P-values were corrected for multiple testing using the FDR method, and only sub-regions with corrected p-values below 0.05 are shown. Beta coefficients from the two hemisphere and from the three metrics were merged, and corresponding hemispheres and metrics for each sub-region are displayed.
