# Supplementary material for: Phenotypic effects of genetic variants associated with autism
Source: Nat Med. 2023 Jun 26;29(7):1671–80. doi: 10.1038/s41591-023-02408-2 (PMC10353945; doi:10.1038/s41591-023-02408-2)
Supplement: Supplementary file 2 — Reporting Summary [file 41591_2023_2408_MOESM2_ESM.pdf]

nature portfolio

Corresponding author(s): Thomas Rolland, Thomas Bourgeron

Last updated by author(s): May 11, 2023

Reporting Summary

Nature Portfolio wishes to improve the reproducibility of the work that we publish. This form provides structure for consistency and transparency in reporting. For further information on Nature Portfolio policies, see our Editorial Policies and the Editorial Policy Checklist.

Statistics

For all statistical analyses, confirm that the following items are present in the figure legend, table legend, main text, or Methods section.

n/a	Confirmed	
☐	☒	The exact sample size (*n*) for each experimental group/condition, given as a discrete number and unit of measurement
☐	☒	A statement on whether measurements were taken from distinct samples or whether the same sample was measured repeatedly
☐	☒	The statistical test(s) used AND whether they are one- or two-sided<br>*Only common tests should be described solely by name; describe more complex techniques in the Methods section.*
☐	☒	A description of all covariates tested
☐	☒	A description of any assumptions or corrections, such as tests of normality and adjustment for multiple comparisons
☐	☒	A full description of the statistical parameters including central tendency (e.g. means) or other basic estimates (e.g. regression coefficient) AND variation (e.g. standard deviation) or associated estimates of uncertainty (e.g. confidence intervals)
☐	☒	For null hypothesis testing, the test statistic (e.g. *F*, *t*, *r*) with confidence intervals, effect sizes, degrees of freedom and *P* value noted<br>*Give P values as exact values whenever suitable.*
☒	☐	For Bayesian analysis, information on the choice of priors and Markov chain Monte Carlo settings
☒	☐	For hierarchical and complex designs, identification of the appropriate level for tests and full reporting of outcomes
☐	☒	Estimates of effect sizes (e.g. Cohen's *d*, Pearson's *r*), indicating how they were calculated

Our web collection on statistics for biologists contains articles on many of the points above.

Software and code

Policy information about availability of computer code

Data collection	We used the Globus v.3.2.0 software to download data from the Simons Foundation for Autism Research Initiatite cohorts SSC and SPARK.
Data analysis	We called the variants using GATK 3.8 for the SSC cohort. We used VEP (using Ensembl 101) to annotate the variants for all cohorts. We visually validated variants with IGV. We used the Admixture software to predict the ancestry of SSC, SPARK and UK-Biobank individuals, based on the 1000genomes reference populations. For autism PGS calculation, we used plink to merge the cohorts and SBayesR with the banded LD matrix to measure the PGS. For the phenome-wide association study, we used the PHESANT v1.1 software. Most of the statistical analyses in this work were performed using statistical test implementations from the scipy and statsmodel python library. Code for post-processing analyses is available at https://github.com/thomas-rolland/subdiagnostic-autism-variants.

For manuscripts utilizing custom algorithms or software that are central to the research but not yet described in published literature, software must be made available to editors and reviewers. We strongly encourage code deposition in a community repository (e.g. GitHub). See the Nature Portfolio guidelines for submitting code & software for further information.

Data

Policy information about availability of data

All manuscripts must include a data availability statement. This statement should provide the following information, where applicable:

- Accession codes, unique identifiers, or web links for publicly available datasets
- A description of any restrictions on data availability
- For clinical datasets or third party data, please ensure that the statement adheres to our policy

Approved researchers can obtain the whole-exome and SNP genotyping data from the SSC and SPARK cohorts used in this study by applying at https://base.sfari.org. The UK-Biobank whole-exome, SNP genotyping, phenotypic and brain imaging data can be obtained by applying at the UK-Biobank database (https://www.ukbiobank.ac.uk/). The human neurodevelopmental transcriptome dataset is available on the BrainSpan database (http://www.brainspan.org). Functional annotations can be obtained from SynGO (https://syngoportal.org/) and Gene Ontology (http://current.geneontology.org/annotations/goa_human.gaf.gz). Human reference genomes were obtained from https://www.ncbi.nlm.nih.gov/grc/human. Electronic health records and healthcare claims data used in the present study for the UK-Biobank individuals are not publicly available due to patient privacy concerns. Prevalence and autism OR measures can be visualized and downloaded on https://genetrek.pasteur.fr/.

Human research participants

Policy information about studies involving human research participants and Sex and Gender in Research.

Reporting on sex and gender	Sex assigned at birth was used for the SPARK sample, and sex from undetermined source for the SSC sample. Genetic sex was used for the iPSYCH sample. Genetic sex was used for the UK-Biobank sample. The fraction of variant carriers was analysed by sex, and sex-specific analysis of autism OR was performed.
Population characteristics	For SSC, SPARK and iPSYCH samples, in addition to rare LoF variant and autism PGS features, we used the sex (see above) and the four first principal components of the PCA based on genotyped SNPs as covariates. For the UK-Biobank sample, in addition to rare LoF variant and autism PGS features, we used the sex (see above), the age, the age-square and the four first principal components of the PCA based on genotyped SNPs for socioeconomic/cognitive analyses, and scanning site for brain imaging analyses.
Recruitment	Recruitment was managed by the SFARI and UK-Biobank consortia. We note that individuals from the UK-Biobank suffer from the "healthy individual bias", that may alter our ability to quantify the actual effect of genetic variants.
Ethics oversight	Informed consents from all individuals were obtained according to following ethics clearances. The Simons Simplex Collection is a multisite effort gathering 12 recruitment sites, informed consents were obtained from all participants included in each site at the time of their initial enrollment and centralized by the Columbia University IRB under the protocol AAAC6306(M00Y17). All SPARK participants were recruited under a centralized IRB protocol (WCG IRB Protocol no. 20151664) and provided written informed consent to take part in the study. Participants of the UK Biobank study provided informed consent and ethical approval was provided by the UK's National Health Service, National Research Ethics Service (Ethics Committee reference number: 11/NW/0382). Data analyses have been conducted in accordance to following research projects that have been deemed exempt under 45 CFR 46.104.d) (4) (ii) by Institut Pasteur IRB: IRB-DB_2019-01 (SSC cohort), IRB2020-K-Exempt (UK Biobank), and IRB-DB_2019-03 (SFARI). The authors confirm that the manuscript complies with current policies on vulnerable groups, and uses current language related to autism.

Note that full information on the approval of the study protocol must also be provided in the manuscript.

Field-specific reporting

Please select the one below that is the best fit for your research. If you are not sure, read the appropriate sections before making your selection.

☒ Life sciences ☐ Behavioural & social sciences ☐ Ecological, evolutionary & environmental sciences

For a reference copy of the document with all sections, see nature.com/documents/nr-reporting-summary-flat.pdf

Life sciences study design

All studies must disclose on these points even when the disclosure is negative.

Sample size	We used all samples from the SSC, SPARK and UK-Biobank for which whole-exome sequencing and SNP arrays were available and predicted from European ancestry, for a total of 226,649 individuals. We also downloaded the variants identified by the Autism Sequencing Consortium in 10,025 individuals. Finally, we filtered out the variants identified among the 44,779 european non-neuro individuals from gnomAD. This is, to our knowledge, the largest study of this kind to date.
Data exclusions	SSC, SPARK and UK-Biobank individuals that did not match our criteria for European ancestry were removed from the analysis. Three families from the SSC cohorts were filtered out due to a high number of erroneous variant calls. Nine families from the SPARK cohort were filtered out due to withdrawal from the cohort. In the UK-Biobank cohort, 3,395 individuals were removed because the corresponding SNP arrays did not

pass our quality control and 198 individuals were withdrawn from the original dataset, 134 individuals were removed because they reported ASD-related symptoms, and 12 because they were twins.

Replication

We replicated the effect of S-LoFs in autism or constrained genes on autism status in one independent analysis of the iPSYCH sample. The replication analysis involved additional quality control steps and different filtering of genetic variants, and provided similar results as the analysis based on SSC and SPARK cohorts.

Randomization

To improve the robustness of our estimation of the odds ratio for autism, we used a sub-sampling procedure iteratively and randomly selecting as many undiagnosed individuals as diagnosed individuals, 100 times. The selection of a sub-sample of undiagnosed individuals was random to reflect the entire population of undiagnosed individuals. The robustness of the p-value associated to the autism-OR was assessed using a bootstrapping procedure, shuffling the labels between diagnosed and undiagnosed individuals, 10,000 times.

Blinding

The calling and quality control of genetic variants in all samples were blind from the phenotype of the individuals. Data collection was blind from genetic variants and polygenic scores for all diagnosed and undiagnosed individuals. For the association studies among undiagnosed individuals, we blindly tested more than 18,000 phenotypes.

Reporting for specific materials, systems and methods

We require information from authors about some types of materials, experimental systems and methods used in many studies. Here, indicate whether each material, system or method listed is relevant to your study. If you are not sure if a list item applies to your research, read the appropriate section before selecting a response.

Materials & experimental systems

n/a	Involved in the study
☒	☐ Antibodies
☒	☐ Eukaryotic cell lines
☒	☐ Palaeontology and archaeology
☒	☐ Animals and other organisms
☒	☐ Clinical data
☒	☐ Dual use research of concern

Methods

n/a	Involved in the study
☒	☐ ChIP-seq
☒	☐ Flow cytometry
☒	☐ MRI-based neuroimaging
